# Phylogeographic and phenotypic divergence between two subspecies of *Testudo graeca* (*T. g. buxtoni* and *T. g. zarudnyi*) across their contact zone in Iran

**DOI:** 10.1038/s41598-022-17926-1

**Published:** 2022-08-09

**Authors:** Neda Ranjbar, Mansoureh Malekian, Mohammad Reza Ashrafzadeh, Mahmoud-Reza Hemami

**Affiliations:** 1 Department of Natural Resources, Isfahan University of Technology, Isfahan, 8415683111 Iran; 2 Department of Fisheries and Environmental Sciences, Faculty of Natural Resources and Earth Sciences, Shahrekord University, Shahrekord, 8818634141 Iran

**Keywords:** Conservation biology, Ecological genetics, Evolutionary ecology

## Abstract

Contact zones are considered as windows into the evolutionary process, allowing identification of factors influencing the evolutionary forces. Here, we combined phylogenetic and morphometric analyses to explore the evolutionary process affecting the taxonomic pattern of two subspecies of *Testudo graeca* (*T. g. buxtoni* and *T. g. zarudnyi*) across their contact zone in Central Iran. Our results showed high levels of phylogeographic and phenotypic variation in the contact zone. Two monophyletic clades including, clade 1 (*T. g. zarudnyi*) and clade 2 (*T. g. buxtoni*) were identified. Furthermore, four distinct subclades were found in *T. g. buxtoni,* across a wide geographic range. Divergence time analysis suggests that the two subspecies diverged from one another after the uplifting of the Zagros Mountains during the early Pliocene. Using neutrality tests and mismatch distribution analysis, we found no evidence of recent population expansion. Morphological associations among geographical populations in the contact zone found more distinctions, with some significant adaptive and non-adaptive morphological variations in these populations. These distinctive morphological populations can be considered as management units (MUs) to conserve the evolutionary potential of this species. Finer scale evolutionary studies are required to address the southern part of the Zagros mountain range, where the overlapping of mitochondrial clades and subclades has occurred. Such information is essential for effective conservation of *T. graeca* populations, preventing translocation or mixing of individuals without comprehensive genetic and morphological assessment.

## Introduction

Contact zones can serve as windows into the evolutionary process that enable us to identify factors influencing the evolutionary forces. The extent and nature of barriers are best understood where divergent taxa are in contact^1^. Therefore, evolutionary biologists are interested in contact zones of distinct taxa because they can provide natural laboratories for assessing hybridization and isolation mechanisms^2^. Moreover, molecular and morphological data of taxa in contact zones elucidate the taxonomic status of a species^1^.

The spur-thighed tortoise (*Testudo graeca,* Testudinidae) is a widely distributed species with pronounced genetic and morphological differentiation between geographically vicariant populations^3–6^. Molecular studies using mtDNA markers have detected several divergent clades, corresponding to the currently recognized subspecies, with admixed populations along contact zones^3,4^. Five more divergent *T. graeca* subspecies exist in the Near and Middle East and southeastern Europe: *T. g. ibera*, *T. g. terrestris*, *T. g. buxtoni*, *T. g. zarudnyi*, *T. g. armenaica*, and *T. g. anamurensis*. Additional monophyletic groups of subspecies have been recognized in the western Mediterranean: *T. g. graeca*, *T. g. cyrenaica*, *T. g. marokkensis*, *T. g. nabeulensis, T. g. soussensis*, and one undescribed lineage. Although several studies have been conducted to evaluate the genetic diversity and population structure within the eastern subspecies^5,7–9^, phylogenetic relationships of these subspecies are not fully resolved^10^. Compared to genetic investigations^3,4,6,11,12^, morphological studies found much more differentiation, suggesting up to 20 distinct taxa within the *T. graeca* complex. More taxa were recognized in the Near and Middle East and southeastern Europe, which were regarded by some authors as distinct species^13–16^. Previous morphological studies focused on traditional morphometrics^4,6,9,17,18^, whereas geometric morphometrics has not been used for shell morphology before.

The spur-thighed tortoise is listed in Appendix II of the Convention on International Trade in Endangered Species of Wild Fauna and Flora (CITES); on the IUCN Red List, the species is assigned to Vulnerable category^19^. This species is threatened by several factors, such as habitat destruction, overharvesting of eggs and adults by local communities, and overharvesting for the pet trade^20–24^. Unrecorded releases of individuals from different lineages may result in the loss of genetic diversity due to genetic erosion^25^. Due to low dispersal ability, strong adaptation to local environmental conditions, and wide geographic distribution^3,4,7^, the development of effective conservation strategies, such as reintroduction or repatriation of individuals into their natural habitat, can be very challenging.

In Iran, three subspecies are currently recognized^26^: (1) *T. g. buxtoni*, distributed from north-western to the center of Iran, (2) *T. g. armeniaca* from north-western Iran, and (3) *T. g. zarudnyi* occurring in the central and eastern parts of the country. The contact zone of *T. g. buxtoni* and* T. g. zarudnyi* is along the Zagros and Irano-Turanian ecoregion in Central Iran^27^. Despite the extensive morphological variability of *T. graeca*, the phylogenetic relationships of Iranian subspecies have remained unknown. This is especially true for the two eastern subspecies, *T. g. buxtoni* and *T. g. zarudnyi*. The current study aimed to (1) fill the phylogeographic gaps of the eastern subspecies, in the extent of *T. graeca* complex, and determine the divergence time of mitochondrial lineages (2) assess the recent population expansion of *T. g. buxtoni* and *T. g. zarudnyi*; (3) clarify morphological and mitochondrial breaks in Central Iran, which is considered to be the contact zone of the two subspecies.

## Results

### Mitochondrial DNA: genetic diversity and phylogeographic analyses

**Figure 1:**
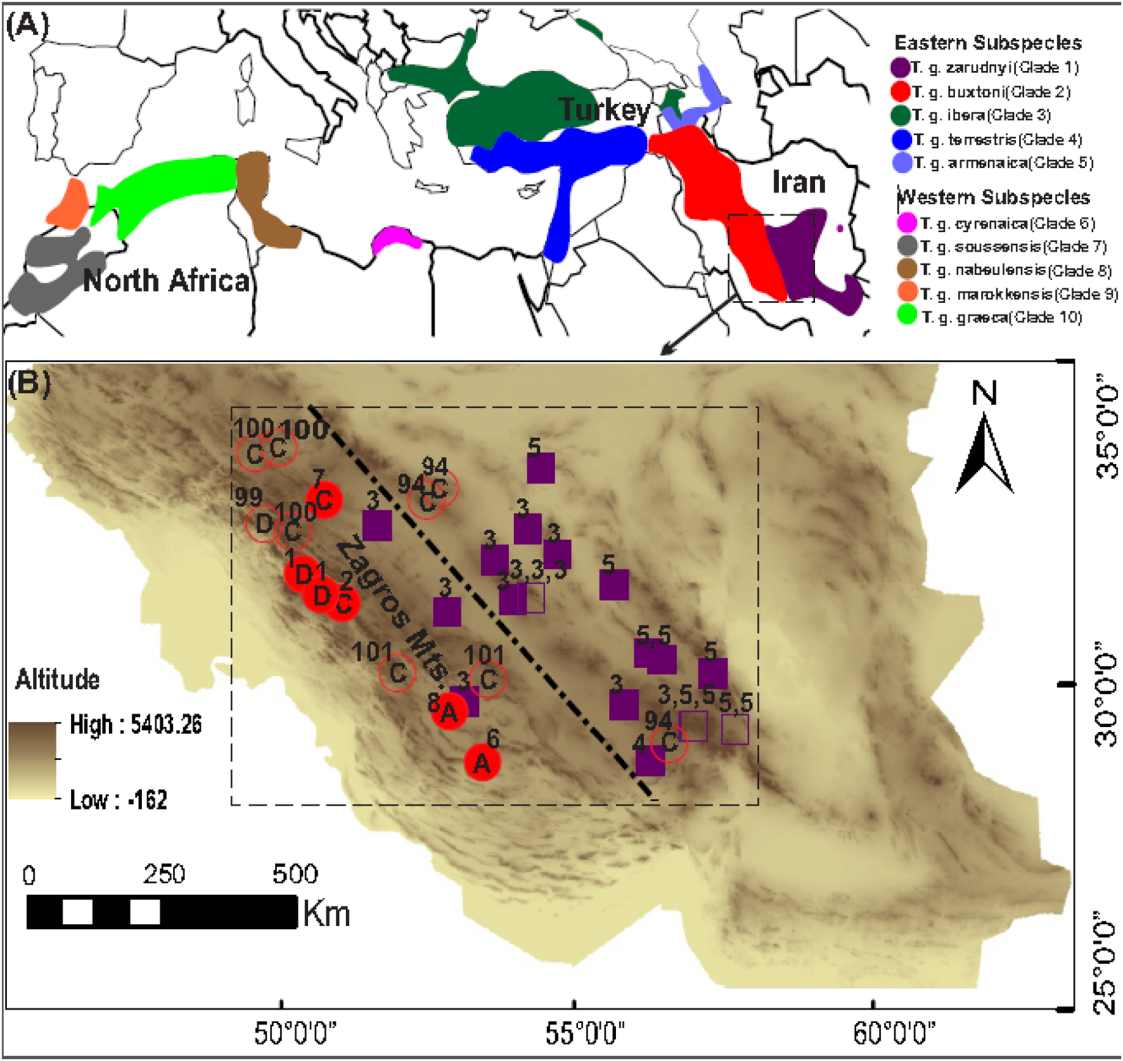
(**A**) The approximate distribution of spur-thighed tortoise’s (*Testudo graeca*) subspecies according to Graciá et al.^10,28^, Turkozan et al.^9^, and Javanbakht et al.^7^. (**B**) Geographical distribution of the sampling sites of the spur-thighed tortoise in the contact zone between two subspecies *T. g. zarudnyi* (Clade 1) and *T. g. buxtoni* (Clade 2) in Central Iran. Purple squares and red circles indicate clades 1 and 2, respectively, and the numbers next to the symbols indicate the haplotypes detected in each sampling sites, according to the numbering assigned to them (see Figs. 2, 3 for details). A, C, and D, denote three subclades found in clade 2 from the study area. Outlined symbols show the sampling sites of the species in Javanbakht et al.^7^. The dashed line refers to the approximate position of the contact zone between the two clades. The base map was obtained from OpenStreetMap contributors, available under Open Data Commons Open Database License (www.openstreetmap.org/copyright) and developed in ArcGIS 10.5.

Phylogenetic status of Iranian spur-thighed tortoises from the contact zone between *T. g. buxtoni* and *T. g. zarudnyi* was evaluated, relative to other subspecies of the species’ global range (Fig. 1), by sequencing a 948 bp fragment of the mitochondrial cytochrome *b* gene. In total, 37 sequences of the two Iranian subspecies were obtained. These sequences were aligned with 229 published spur-thighed tortoise cytochrome *b* sequences, retrieved from GenBank. See Table S1 for a complete list of sequences and corresponding haplotypes used in the present study. All new sequences were submitted to GenBank (accession numbers: ON887288-ON887307).

**Figure 2:**
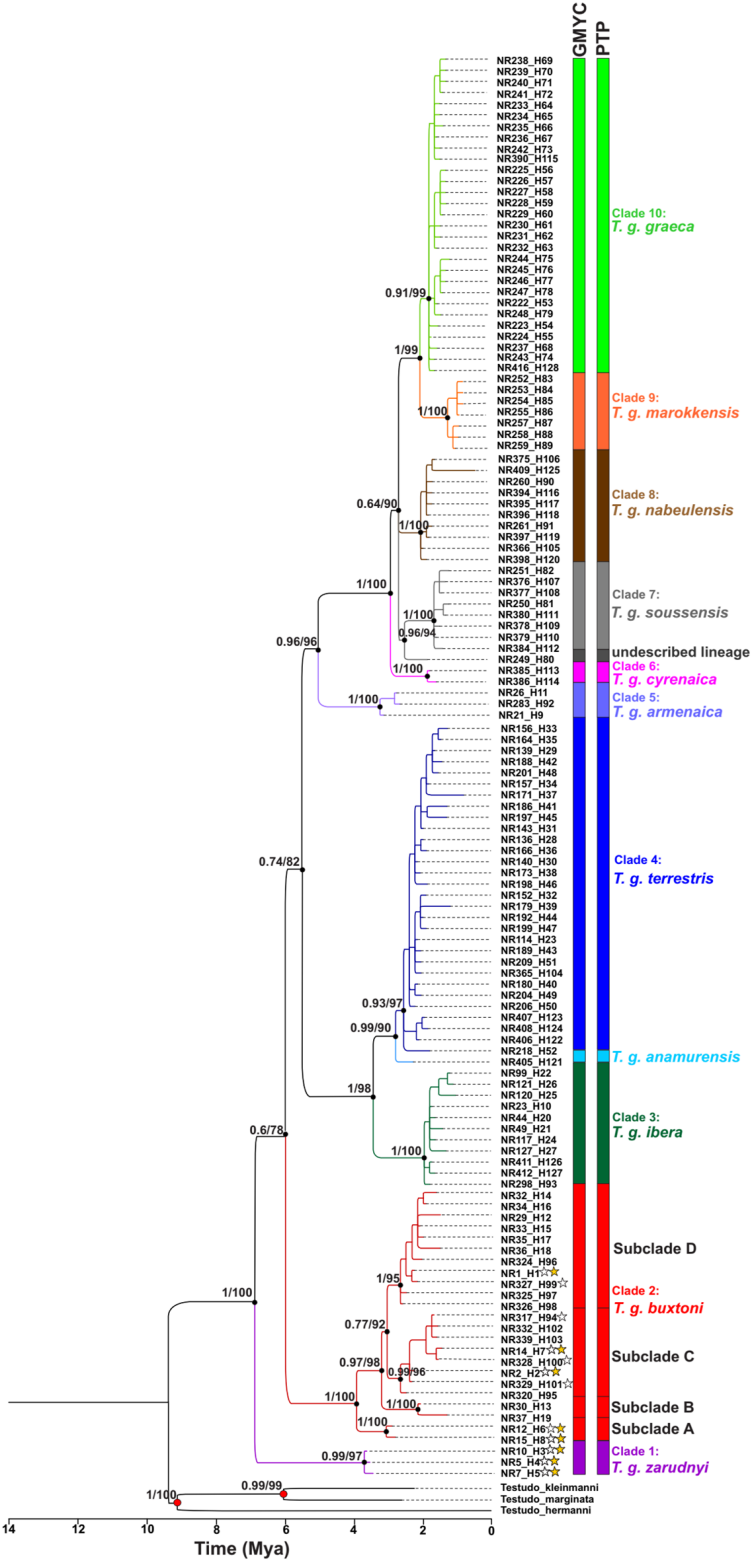
Phylogenetic relationships of *Testudo graeca* based on the 948-bp mtDNA cyt *b* gene sequences and rooted with *T. kleinmanni* (AJ888371), *T. marginata* (AJ888333), and *T. hermanni* (AJ888362). The identical topology derived from Bayesian inference (BI) summarized with 50% majority rule consensus tree and the best tree of maximum likelihood (ML) with species delimitation analyses using Poisson Tree Processes (PTP) and General Mixed Yule Coalescent (GMYC) methods. The numbers on nodes represent posterior probabilities in BI and bootstrap values in ML, respectively. The main clades recognized as subspecies according to Fritz et al.^3,4^ and Mashkaryan et al.^8^. Stars indicate haplotypes detected in the contact zone between clades 1 and 2.

Based on 948 base pairs of cytochrome *b* from 336 mtDNA sequences, 128 haplotypes, including five new haplotypes from Iran, were identified. Analyses of maximum likelihood (ML) and Bayesian inference (BI) yielded identical tree topologies in which 10 lineages from the species’ worldwide range (Fig. 2) were recognized. Spur-thighed tortoises were divided into eastern Mediterranean (clades 1 to 5) and western Mediterranean (clades 6 to 10) lineages.

In the contact zone, two monophyletic clades: clade 1 (subspecies *T. g. zarudnyi*), and clade 2 (subspecies *T. g. buxtoni*) were identified. Clade 1 is predominantly distributed throughout the eastern part of the contact zone, whereas clade 2 is mainly distributed in the western part of the contact zone with an overlap across the central part of the contact zone (Fig. 1). Clade 2 is further subdivided into four subclades (A, B, C, and D, Fig. 2), three of which (A, C, and D) were found in Iran, whereas subclade B was only identified in sequences from Turkey. The subclade C is the most geographically wide-spread group in the western part of the contact zone, however, the other two subclades (A and D) are restricted to small geographic areas (Fig. 1) (Table 1).

**Table 1:** Genetic diversity statistics for *Testudo graeca* in the contact zone between clade 1 (*T. g. zarudnyi*) and clade 2 (*T. g. boxtoni*).

Phylogenetic groups	N	S	H	h ± SD	π ± SD	K	R_2_	P (R_2_)	D	P (D)	F_s_	P (F_s_)	r	P (r)
Clade 1 (*T. g. zarudnyi* )	22	3	3	0.558 ± 0.06	0.0012 ± 0.00	1.10	0.16	0	− 0.12	0.57	0.45	0.443	0.09	0
Clade 2 (*T. g. buxtoni* )	15	33	9	0.924 ± 0.04	0.0113 ± 0.00	10.74	0.14	0	− 0.14	0.57	0.15	0.48	0.06	0
Total	37	49	12	0.835 ± 0.04	0.0158 ± 0.00	15.02	0.11	0	− 0.09	0.56	− 0.14	0.52	0.02	0

N, Number of individuals; S, Number of polymorphic sites; H, Number of haplotypes; h, Haplotype diversity; π, Nucleotide diversity; K, The average number of pairwise differences; R_2_, Ramos-Onsins and Rozas’s test; D, Tajima’s test; FS, Fu’s test; r, raggedness index. P (R_2_), P (D), P (FS), and P (r) are *p* values of the statistical tests.

**Figure 3:**
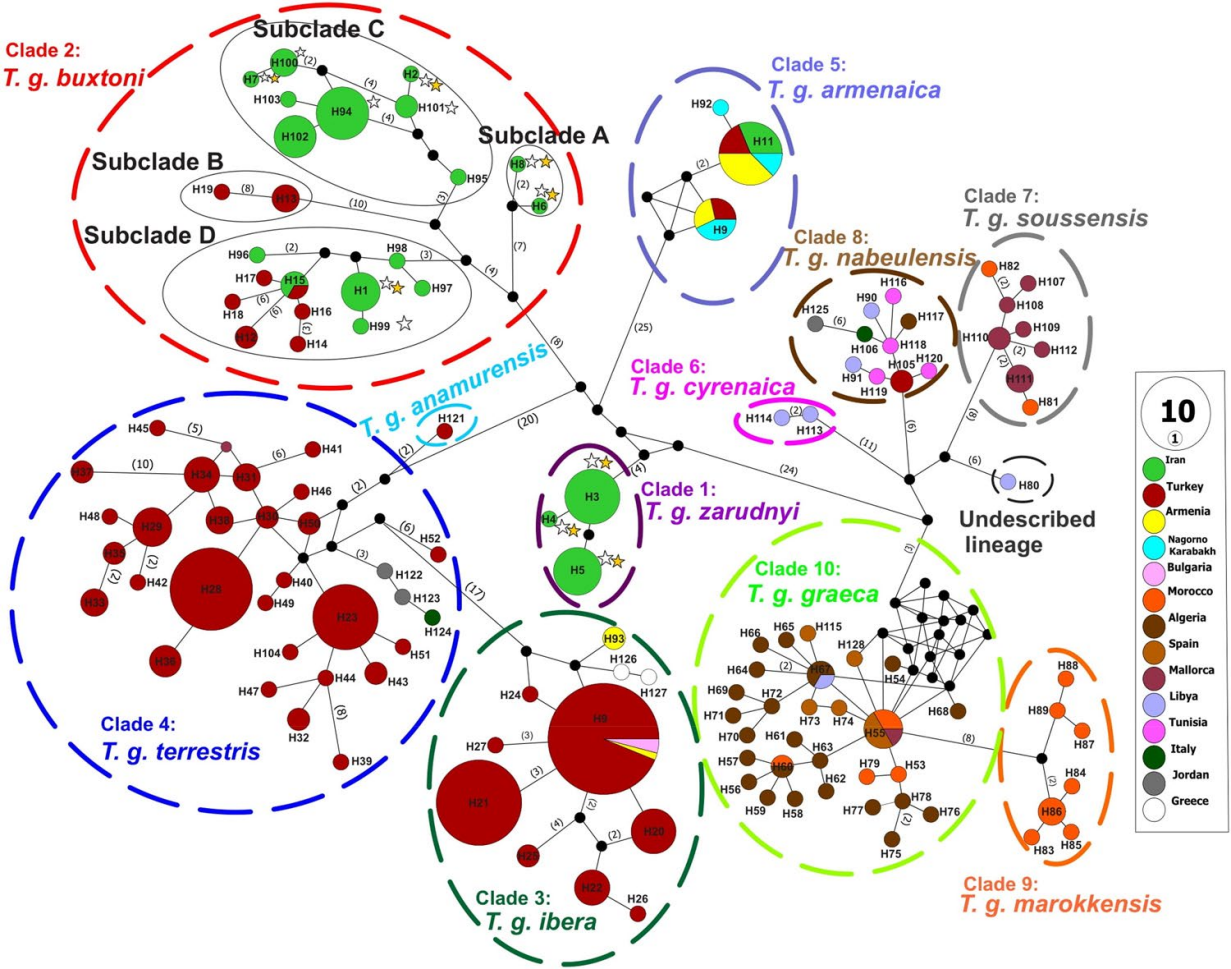
Median-joining network of *Testudo graeca* based on the 948 bp mtDNA cyt b gene sequences. The sizes of the circles represent the haplotype frequency, and colors indicate the country of origin. The groups of haplotypes corresponding to the clades and subclades in Fig. 2 are bounded by ovals with dashed lines. Numbers next to the connecting lines are appeared when the mutational steps among haplotypes are > 1. Small black circles depict inferred missing haplotypes. Stars indicate haplotypes detected in the contact zone of clades 1 and 2. Open stars indicate the haplotypes reported by Javanbakht et al.^7^.

A median joining (MJ) network, illustrating the relationship between spur-thighed tortoise haplotypes, is depicted in Fig. 3. The median joining (MJ) network analysis (Fig. 3) supported the 10 lineages distinguished by the phylogenetic trees. In total, 12 haplotypes were identified among the specimens in the contact zone, including three (H3-H4-H5) and nine (H1-H2-H6-H7-H8-H94-H99-H100-H101) haplotypes in clades 1 and 2, respectively. Within the clade1 (*T. g. zarudnyi*), H3 and H5 were the most common haplotypes that were shared by 12 and 9 individuals, respectively (Fig. 1) and only new haplotype H4 was detected in one individual from southeast of the contact zone. In contrast, four new haplotypes (H2, H6, H7and H8) were detected in clade2 (*T. g. buxtoni*).

In an analysis of molecular variance (AMOVA), significant F_ST_ was obtained among populations, with 92% of the variation was partitioned among populations and 8% within populations. The significant genetic structure was proved by high fixation index (F_st_ = 0.92) (Table 2).

**Table 2:** AMOVA results for *Testudo graeca* populations in the contact zone of *T. g. zarudnyi* and *T. g. boxtoni* based on the 948 bp of cytochrome *b* gene sequences.

Source of variation	d.f	Percentage of variation	Fixation index	*p* value
Among populations	3	92.4	F_st_ = 0.92	*P* < 0.000
Within populations	33	7.6		
Total	36			

#### Molecular clock estimates

Divergence times of all supported nodes (> 0.5/50) in the phylogenetic tree are given in Table 3. The divergence time for *T. graeca* in Iran was averaged at about 6.7 Mya with 95% highest posterior density (HPD) intervals of 5–8.4. The divergence time of *T. g. zarudnyi* was estimated at about 3.9 Mya (95% HPD: 2.3–5.5), while the value for *T. g. buxtoni* was about 4 Mya (95% HPD: 2.7–6.9).

**Table 3:** Molecular clock estimations of *Testudo graeca*.

Node (split between)	Node age (Mya)	95% highest posterior density intervals (HDP)
*T. graeca*	6.7	5–8.4
*Clade 1* (*T. g. zarudnyi*)	3.9	2.3–5.5
*Clade 2* (*T. g. buxtoni*)	4.2	2.7–6.9
Subclade A	3.2	1.9–4.6
Subclade B	2.2	1.2–3.4
Subclades C + D	3.2	2.3–5.5
*T. g. ibera* and *T. g. terrestris*	3.8	2.3–5.6
*T. g. armenaica* and Western Mediterranean lineages	5.5	3.7–7.2

See Fig. 2 for details of clades and subclades.

#### Demographic analysis and neutrality tests

**Figure 4:**
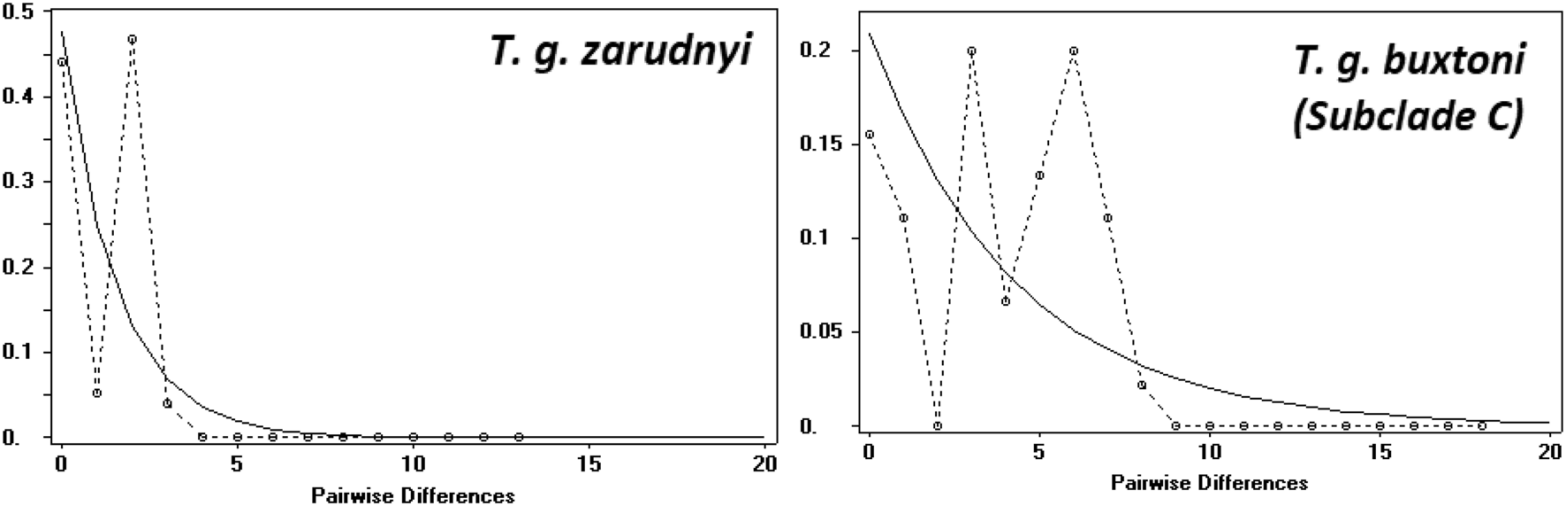
Demographic history of *T. g. zarudnyi* and* T. g. buxtoni* (subclade C) represented by mismatch distribution analyses; observed (dashed line) and expected (solid line).

Mismatch distribution analyses and neutrality tests suggested no evidence for unimodal distribution and didn’t reveal significant population expansion (Fig. 4).

### Traditional morphometric analyses

**Figure 5:**
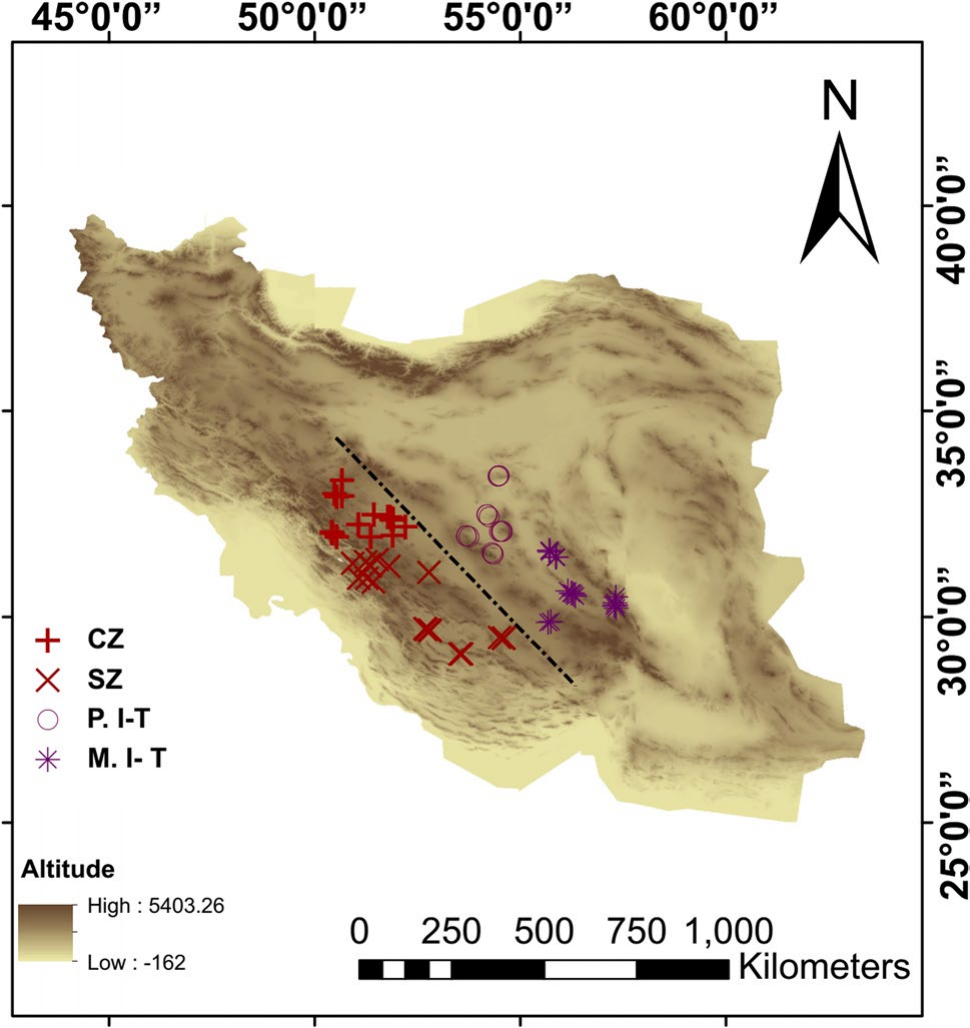
Four morphological clusters of *Testudo graeca* in the contact zone of *T. g. zarudnyi* and* T. g. buxtoni* in the Central Iran. CZ: Central part of Zagros Eco-region, SZ: Southern part of Zagros Eco-region, P. I-T: Plain areas of Irano-Turanian Eco-region and M. I-T: Mountainous areas of Irano-Turanian Eco-region. The base map was obtained from OpenStreetMap contributors, available under Open Data Commons Open Database License (www.openstreetmap.org/copyright) and developed in ArcGIS 10.5.

The cluster analysis revealed the presence of four distinct morphological clusters across the study area (Fig. 5). When the number of clusters was changed, the degree of similarity significantly decreased and the distance increased. Therefore, the optimal number of clusters was four (Table 4). The sampling localities were grouped into four populations, including Central part of Zagros Eco-Region (CZ), Southern part of Zagros Eco-Region (SZ), Plain areas of Irano-Turanian Eco-Region (P.I-T) and Mountainous areas of Irano-Turanian Eco-Region (M.I-T) (Fig. 5).

**Table 4:** Number of morphological clusters, similarity and distance levels of *T. graeca* across the study area.

Steps	No. clusters	Similarity level	Distance level	Clusters joined	New cluster	No. observation in new cluster
77	10	89.34	0.42	74–82	74	14
78	9	88.62	0.45	1–7	1	61
79	8	88.42	0.45	12–64	12	6
80	7	87.50	0.49	1–12	1	67
81	6	86.51	0.53	13–26	13	3
82	5	85.77	0.56	1–61	1	68
83	4	82.17	0.698	1–41	1	69
84	3	74.96	0.980	1–13	1	72
85	2	73.74	1.03	1–3	1	73
86	1	41.54	2.29	1–74	1	86

**Figure 6:**
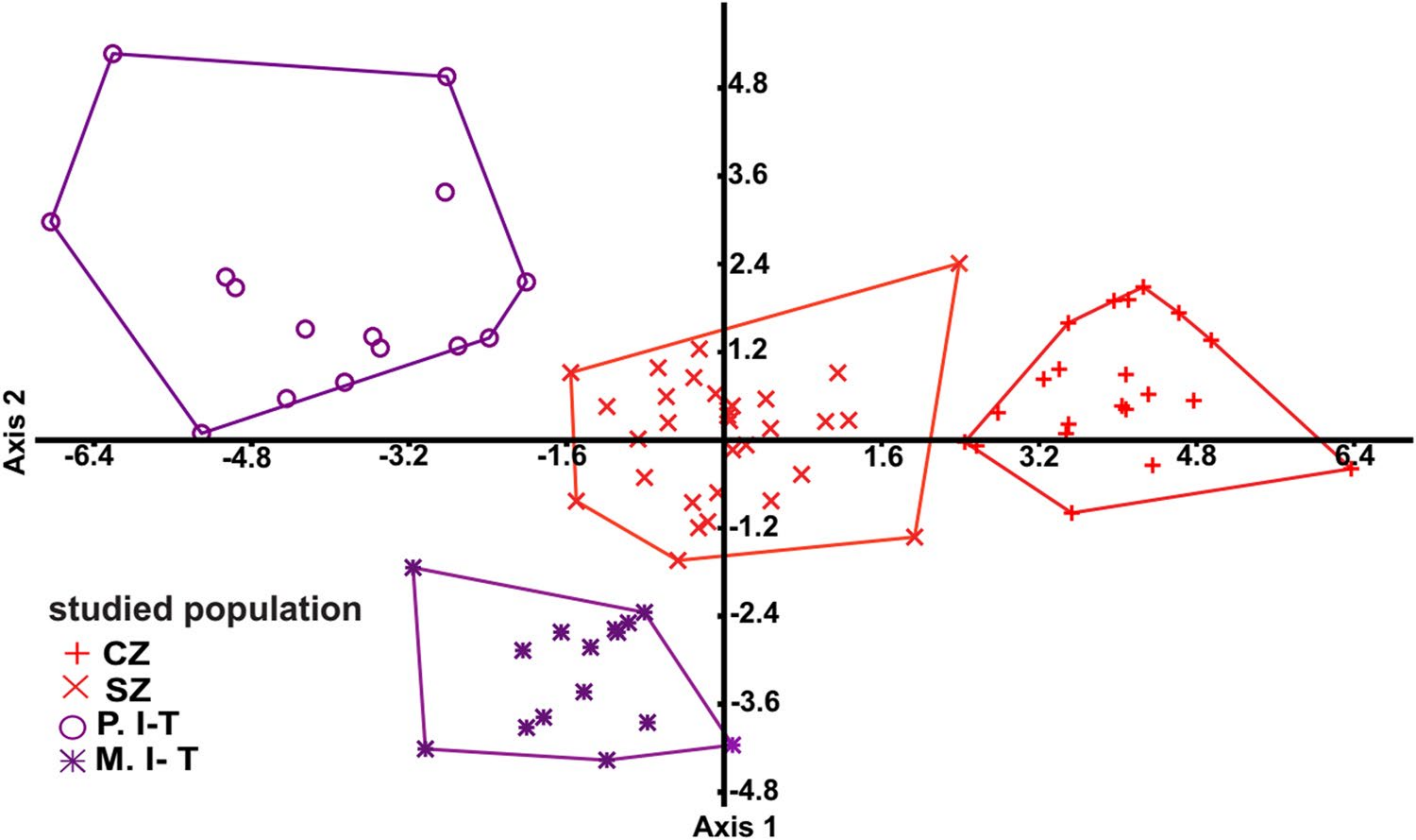
Scatter plot of the two first canonical axes resulted from canonical variate analysis, showing the distribution of *T. graeca* populations in Central Iran.

Canonical and multivariate analyses of variance showed a significant difference between the body shape of the studied populations (*P* < 0.0001) (Fig. 6). Results of discriminant analysis (DA) indicated that 95% of the sampled individuals were classified into the correct group. Based on the squared distance between the studied groups, the populations were mostly related to the same subspecies as expected (Table 5). Further, multiple regression analysis confirmed that CZ and M.I-T have the highest regression coefficients and are quite different from other groups (Table S3). Based on the regression coefficients, variables with the highest coefficients include MCW, MGSL, AbSL, FSL, NW, and IHASO. Some variables such as MCW, FSL, and IHASO showed greater coefficient in CZ and SZ, while MGSL, AbSL, and NW had greater coefficient in M.I-T and P.I-T. Variables including CH, CHSW, CFSW, CSAW, GSL, VW1, VW2, VW3, VW5, CL1, CL2, and CL3 showed greater coefficient in CZ and M.I-T, compared to SZ and P.I-T. The difference between M.I-T and P.I-T was strongly related to PSL, NL and SL, as PSL has the highest coefficient in M.I-T, while NL and SL showed the highest coefficient in P.I-T.

**Table 5:** Squared distance among studied populations of *T. graeca* in Central Iran, using discriminant function analysis.

	CZ	SZ	P. I-T	M. I-T
CZ	2.854	18.848	32.806	33.992
SZ	19.714	1.989	20.187	19.359
P.I-T	32.442	18.956	3.219	25.464
M.I-T	33.525	18.026	25.362	3.321

Population codes are given in Fig. 5.

### Geometric morphometric analyses

**Figure 7:**
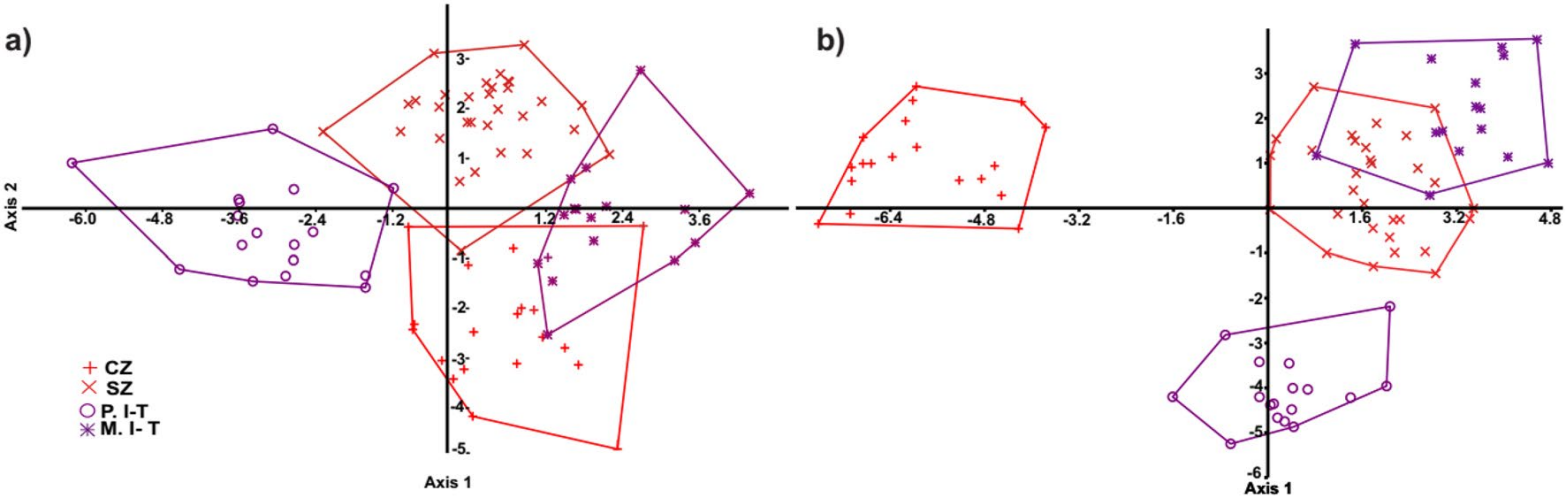
Scatterplot of the two first canonical axes of carapace (**a**) and plastron (**b**) shape variation, showing the distribution of *T. graeca* populations in Central Iran.

In order to assess the shape changes of the carapace and plastron, their TPS files were imported separately into PAST and Morpho J software. Three CVA components were extracted. The first and second components accounted for 79.2% and 77.4% of variation in carapace and plastron, respectively. The *P* values (*P* < 0.0001) from permutation tests (10,000 permutation rounds) indicated significant Mahalanobis distances among studied populations (Table 6). In plastron shape data, overlap was observed between M.I-T and CZ populations, while no overlap was observed in CZ and P.I-T (Fig. 7a). Based on the carapace data, only M.I-T and SZ populations showed overlap (Fig. 7b). The most distinct population based on carapace shape variation was CZ, while the maximum Mahalonobis distance was observed between P.I-T and other populations, based on the plastron shape variation.

**Table 6:** Mahalanobis distances of carapace and plastron shape variation among *T. graeca* populations in Central Iran.

	Code	CZ	SZ	P.I-T
Carapace	SZ	7.7545		
	P.I-T	7.8712	5.8574	
	M.I-T	9.2587	5.7611	7.1398
Plastron	SZ	5.0693		
	P.I-T	5.9488	6.8814	
	M.I-T	4.2452	5.9091	4.9119

Population codes are given in Fig. 5.

**Figure 8:**
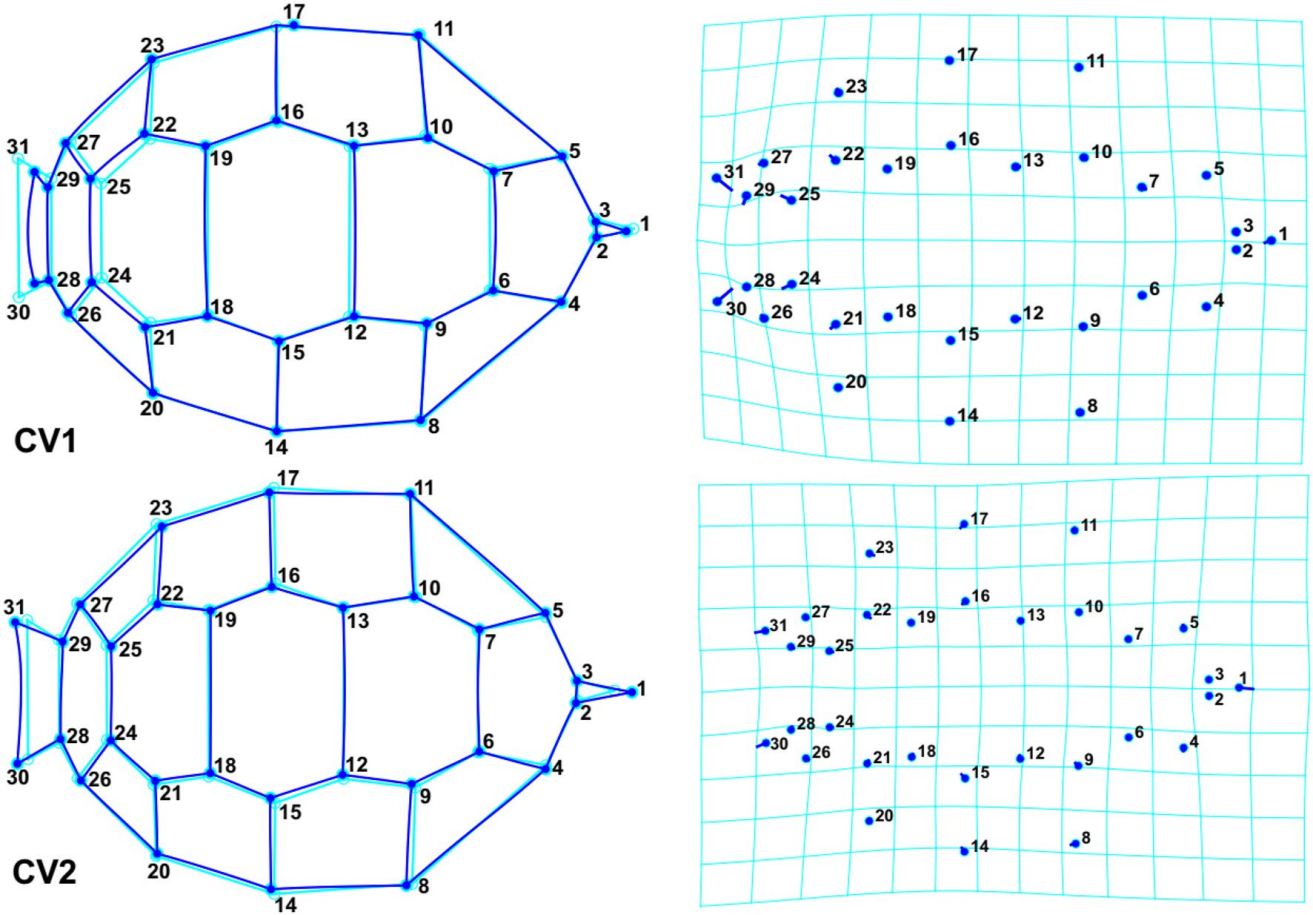
Carapace shape variation of *T. graeca* populations defined by transformation grids (right) and wireframe graphs (left) of the first two canonical axes in Central Iran.

**Figure 9:**
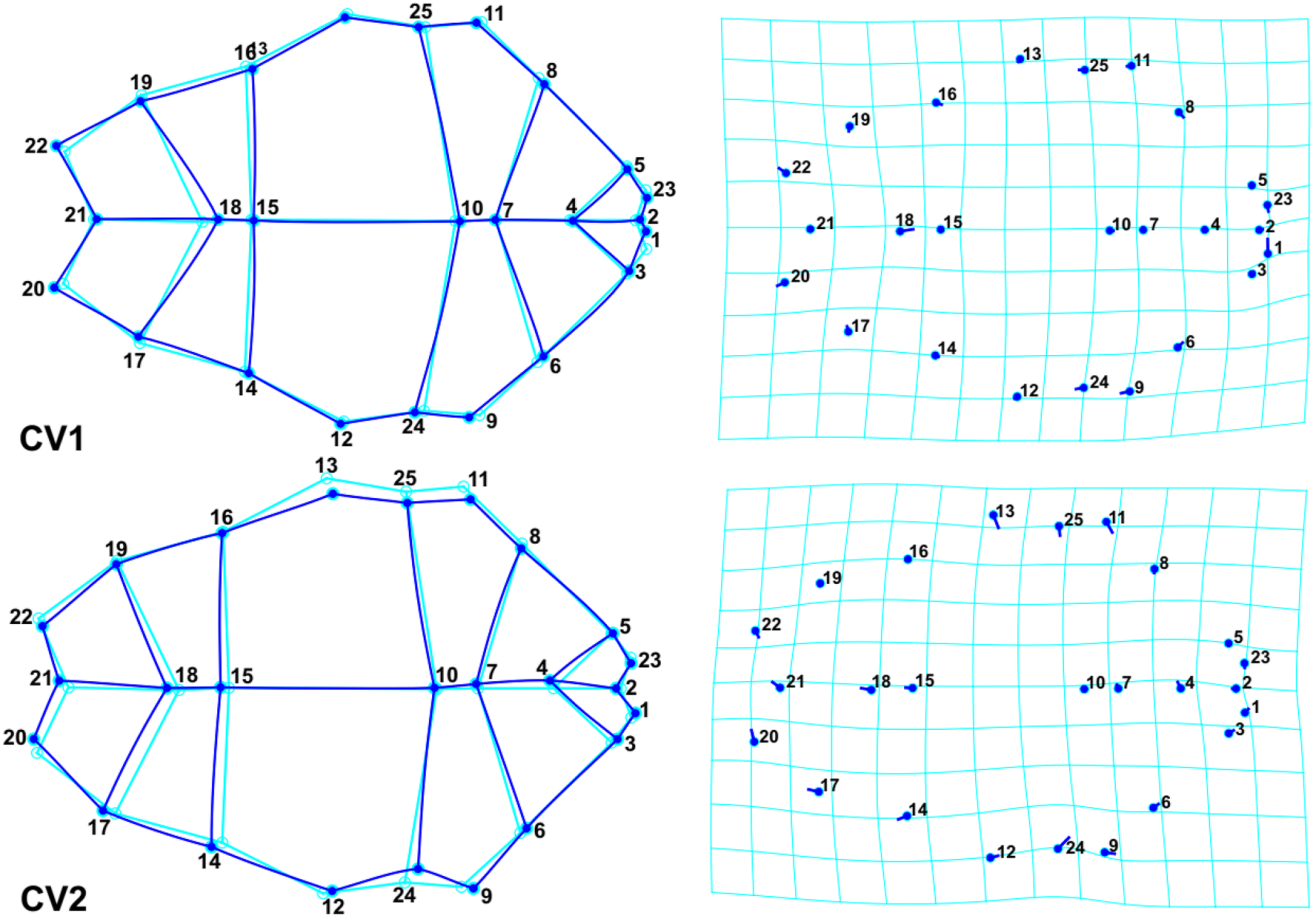
Plastron shape variation of *T. graeca* populations defined by transformation grids (right) and wireframe graphs (left) of the first two canonical axes in Central Iran.

**Figure 10:**
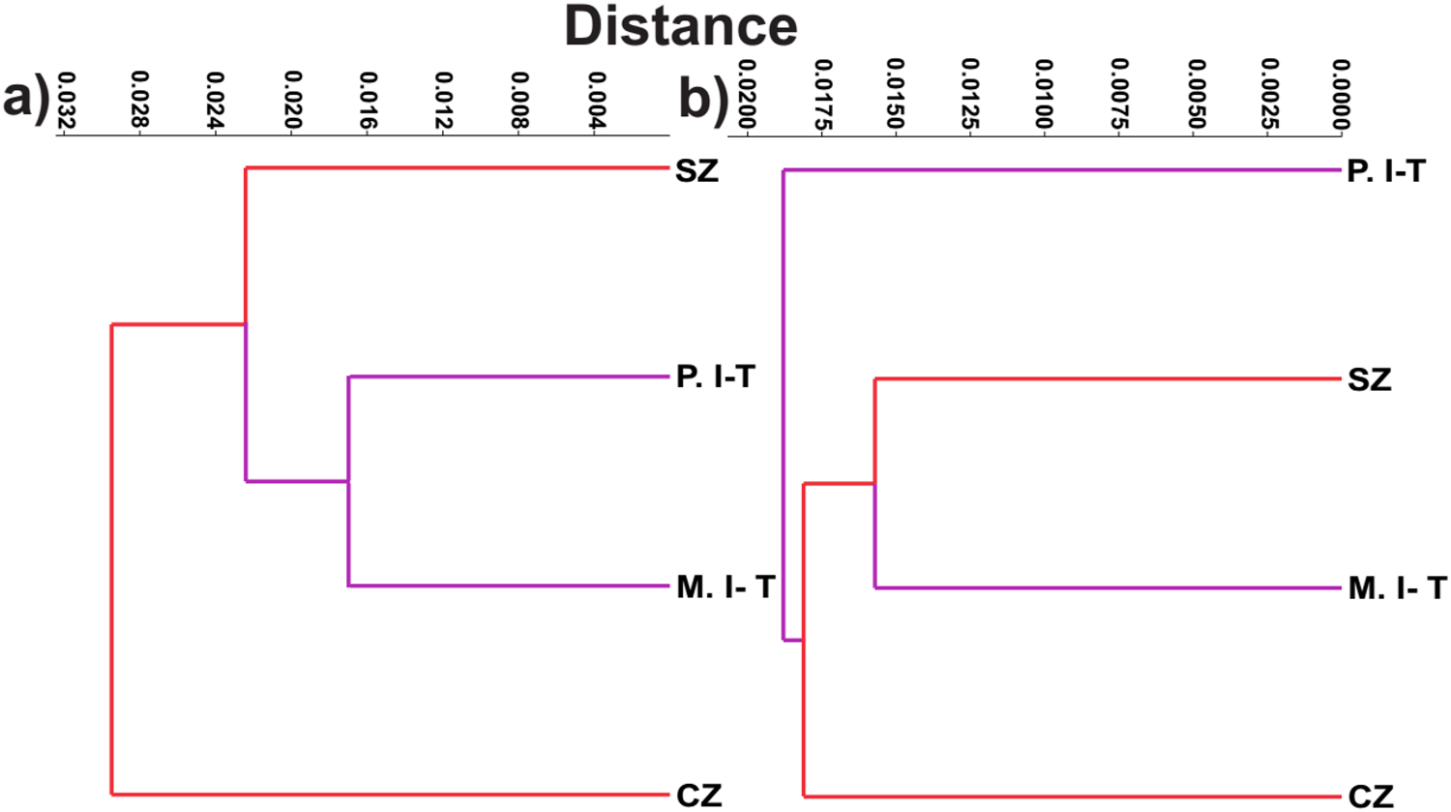
Cluster dendrogram of *T. graeca* populations in Central Iran, based on carapace (**a**) and plastron (**b**) shape variations.

To further illustrate shape changes, transformation grids and wireframe graphs were applied on the carapace (Fig. 8) and plastron (Fig. 9) data. In the first component, the main carapace shape changes were in the supracaudal and fifth vertebral scales, while NL was considerably changed in the second component. The middle landmarks showed the least amount of displacement. The highest displacement, in the first plastron CVA component, was related to landmark No. 18 (connection of femoral and anal sutures). In addition to this landmark, the most movements corresponded to landmark No. 4 in the second component. Cluster dendrograms were constructed based on the consensus of each studied population. The Copernic coefficient of this analysis for carapace and plastron were 0.92 and 0.87 respectively, suggesting the distinction of studied populations based on both carapace (Fig. 10a) and plastron (Fig. 10b).

## Discussion

The phylogeography, morphological diversity, and population structure of the most widely distributed tortoise, *T. graeca* have been investigated by several studies^3,4,6–9,18,29^. However, contact zones among subspecies can raise questions regarding the evolutionary boundaries and admixture^28^. Therefore, the current study concentrated on the contact zone of two early-divergent and endemic subspecies of *T. graeca (T. g. buxtoni* and *T. g. zarudnyi*) in Central Iran to fill phylogeographic and phenotypic gaps in this area. Moreover, the results provide essential information to develop effective conservation plans. The main results are elaborated in the sections below.

### Mitochondrial diversity and phylogeography

Our finding confirmed the general topology suggested by previous studies^3,10^. Incorporating sufficient samples of eastern subspecies into our analyses has led to some achievements in the phylogenetic structure of the species. In our analysis, 10 divergent clades were identified, associating with the current known subspecies of *T. graeca.*

The two subspecies, *T. g. zarudnyi*, and *T. g. buxtoni* formed two monophyletic clades. Four subclades were further identified within *T. g. buxtoni*, three of which (A, C, and D) were found in Iran, whereas subclade B was only identified in sequences from Turkey. The subclade C was the most geographically wide-spread group in the contact zone. However, the other two subclades (A and D) were restricted to small geographic areas. Subclades A and B could probably adapt to their local environment, while subclades C and D (also defined as haplogroups in previous studies^7^) were widely distributed throughout *T. g. buxtoni* geographic range. Subclade D was mainly distributed outside the contact zone, with a high level of genetic diversity. Subclade C was the most diverse and widely distributed haplotype in the contact zone. It shares one haplotype (H94) in the northern and southern parts of *T. g. zarudnyi* distribution range. We found *T. g. buxtoni,* the only subspecies, with distinct subclades in a wide geographic range. Therefore, it is recommended to introduce new subspecies, particularly in western part of the region, which has been pronounced in a considerable genetic diversity and differentiation through a long evolutionary history^9^.

The second subspecies (*T. g. zarudnyi*) had a smaller distribution range in the contact zone, having three haplotypes. Haplotype H5 is distributed further from the two subspecies border and confined to the previously recognized distribution range^7^, while haplotype H3 is the most frequent haplotype in the contact zone. This haplotype was also recorded in some localities in the distribution range of *T. g. buxtoni,* close to the border of the two subspecies.

Divergence time analysis suggests that the two subspecies diverged from one another after the uplifting of the Zagros Mountains during the early Pliocene^30^. The divergence time estimates were coherent with the study of Fritz et al.^3,4^ and Garcia^10^, suggesting that *T. graeca* had spread over the Pliocene, though our dating results were in narrower ranges compared to estimates of the previous studies^10^.

Our results did not support the hypothesis of population expansion of *T. graeca* in the study area, which is in line with the previous study^7^. Therefore, the high genetic diversity as well as divergence, especially in the contact zone, could be the result of long term stability in their distribution ranges^7^.

With our dataset in the contact zone, we obtained high level of genetic diversity and divergence with some expected introgressions. Genetic diversity statistics indicate high values of haplotype and nucleotide diversity, particularly for *T. g. Buxtoni* (Table 1). Clear genetic structure depicted in the phylogenetic tree (Fig. 2) and the haplotype network (Fig. 3), particularly for *T. g. zarudnyi,* was confirmed by F_st_ and an analysis of variance (Table 3). Our analyses showed the presence of divergent clades with no evidence of recent population expansion and frequent and widespread haplotypes (e.g. H94 and H3 as potential ancestral haplotypes) in the contact zone. Therefore, we recognized the contact zone of *T. g. buxtoni* and *T. g. zarudnyi* as an evolutionary significant zone in the distribution range of the species.

### Traditional and geometric morphometrics

Our morphological study is the first to be performed in the contact zone between the two subspecies of *T. graeca* in Iran. We examined a number of morphological variables and identified the most important morphological features pronounced in shapes. We identified four distinct groups in Central part of Zagros Eco-Region (CZ), Southern part of Zagros Eco-Region (SZ), Plain areas of Irano-Turanian Eco-Region (P.I-T) and Mountainous areas of Irano-Turanian Eco-Region (M.I-T). These regions are different based on the most significant climatic factors, including annual precipitation and temperature seasonality (Table S2).

The high discriminatory power of geometric morphometrics^31,32^ was led to differentiate morphological populations, using carapace or plastron landmarks. Our finding is in agreement with the suggestions of population-specific variation, phenotypic and ecological plasticity among populations of tortoises^4,6,33–35^. The greatest morphological distance was found between the Zagros (mainly *T. g. buxtoni*) and Irano- Turanian (mainly *T. g. zarudnyi*) populations. In geometric morphometric analyses, the differentiation among morphological populations was completely clear for CZ in carapace and P.I-T in plastron. Although Copernic coefficients represented the distinctiveness of morphological populations, based on the existing overlaps, some similarities between CZ and M.I-T populations (in plastron) and between SZ and M.I-T (in carapace) were observed. Moreover, SZ was located at the center of the traditional CVA scatter plot (Fig. 6), and in the same group with *T. g. zarudnyi* populations in the cluster analysis (Fig. 10). This implies that some parts of the SZ may have facilitated gene flow among tortoise populations, due to the lack of biogeographical barriers such as mountain ranges and rivers. For example, our cluster analyses suggest CZ and SZ are considerably distinct, possibly due to the presence of the Khersan river. In addition, different environmental conditions in plain and mountainous areas seem to influence the distinction between P. I-T and M. I-T (especially in plastron shape changes). For example, our genetic assessments demonstrate no difference between two morphological populations, P. I-T and M. I-T. This can be explained by the role of phenotypic plasticity in adaptation of the populations to novel environments^4,36–38^.

As it can be inferred from our results, environmental conditions act effectively on costal scales; carapace height, and width of plastron lobes^17,39^. In open areas with limited shelter and food, as is the case in P. I-T, digging ability is positively selected^40^ as a more flatted carapace may improve burrowing. Further, the carapace (CW and particularly MCW) and plastral lobes were narrower in P. I-T populations to provide wider openings for head and limbs and facilitate mobility. By contrast, the height of the carapace, width of vertebral scales and plastral lobes are greater in the populations of CZ and M. I-T and they have longer coastal scales. This can be attributed to the different environmental conditions in CZ and M. I-T compared to P. I-T (e.g. more precipitation and lower temperature seasonality), which can stimulate more plant growth, and the availability of more shelters in mountainous landscapes.

Our results illustrated the importance of supracaudal and nuchal carapace scales for distinguishing populations. Previous studies^39,41^ have also recommended the supracaudal and nuchal scales as reliable diagnostic characters for distinguishing the populations of *Testudo* species. Furthermore, some morphological variables such as MGSL, AbSL, FSL, GSL, PSL and IHASO can be used for discriminating the populations. Among these variables, IHASO is visually more obvious. The decrease in inner height of the anterior shell in P.I-T and M.I-T is evidently due to the inclination of the gular scales inward.

### Combining mitochondrial and morphological data

The separation of the two subspecies is remarkably proved by mitochondrial clades as well as traditional and carapace geometric morphometrics. It seems that the Zagros Mountains act as ecological barriers, separating the geographic ranges of the two subspecies, though geographical overlap between mitochondrial clades and morphological populations in the southern part of Zagros implies the possibilities of gene flow in this part of the contact zone. For example, similar environmental conditions of M. I-T and CZ may have resulted in similar morphological features in the populations of these two subspecies (see above). Based on the current study, we recommend that these distinctive morphological populations can be considered as management units (MUs) to conserve the evolutionary potential causing adaptive and non-adaptive morphological variations, more effectively^42^. These key phylogeographic and phenotypic achievements remind the importance of contact zones as suitable sites for studying evolutionary processes. However, finer scale evolutionary studies are required to address the southern part of the Zagros mountain range, where the overlapping of mitochondrial clades and subclades as well as the morphological populations are happening. Consequently, translocation or mixing of individuals, even for conservational purposes, cannot be acceptable without comprehensive genetic and morphological assessment.

## Materials and methods

### Ethic statement

Samples and data were collected in accordance with the guidelines and regulations of the Iranian Department of Environment (DOE) under permission No. 97/6549. All individuals were sampled, photographed, morphologically measured and released at the point of capture with no harm. Sample collection followed the ethical recommendations in the ARRIVE guidelines^43^. Biopsy sampling technique as a non-destructive, quick and safe method was carried out to reduce the possibility of bleeding and infection^44^. However, alcohol was used before and after the sampling.

### Sampling

The sampling was conducted in two consecutive years (2018 and 2019) during the species activity period in the contact zone of *T. g. buxtoni* and *T. g. zarudnyi* in Central Iran (Fig. 1). A total of 86 individuals were collected from Fars, Kerman, Isfahan, Yazd, Chaharmahal and Bakhtiari and Kohgiluyeh and Boyer-Ahmad provinces. For morphological analyses, however, only 82 adult individuals were measured and photographed. Growth rings on the costal scale of the carapace were counted and individual with more than 12 years old were considered as adults^45^. Tissue samples were taken from individuals covering the contact zone, using a biopsy punch. Small biopsy punches (2 mm) were used to obtain 0.5 mm deep tissues from the scales under feet close to the spurs. The tissue was immediately stored in pure 99% ethanol.

### Laboratory procedures

Genomic DNA was extracted from tissue samples using WizPrep™ gDNA Mini Kit (Cell/Tissue) following the manufacturers’ instructions. Polymerase chain reaction was performed for amplification of a 948 bp fragment of the mitochondrial cyt *b* gene, using CytbG (5′ AACCATCGTTGTWATCAACTAC 3′) and mt-f-na (5′AGGGTGGAGTCTTCAGTTTTTGGTTTACAAGACCAATG 3′) primers^46,47^. Amplifications were performed in 25 µl volumes, containing 10 µl of the Ampliqon PCR master mix, 1 µl of each primer, 9 µl H_2_O and 4 µl of DNA. The thermocycling was performed using an initial denaturation at 95° for 5 min followed by 35 cycles of 30 s at 94°, 50 s at 55°, and 1 min at 72° and a final extension at 72° for 7 min. Purified PCR products were sequenced on an ABI PRISM 3730xl using the BigDye Terminator Cycle Sequencing kit v.3.1 (Applied BioSystems).

### Alignment and sequence analyses

Sequences were edited with SeqScape v.2.6 software (Applied Biosystems) and aligned with 229 previously published mtDNA sequences from GenBank using the ClustalW algorithm implemented in Mega v.7^48^ and checked visually. In addition to *T. g. buxtoni* and *T. g. zarudnyi* sequences, all other subspecies sequences were retrieved from GenBank and included in the final dataset. Sequences of *T. hermanni* (AJ888362), *T. marginata* (AJ888333), and *T. kleinmanni* (AJ888371) were used as outgroup representatives.

The number of polymorphic sites (S), nucleotide diversity (π), number of haplotypes (H), haplotype diversity (h), and the average number of pairwise differences (K) were calculated using DnaSP 5.10.1^49^. The substitution saturation was checked in DAMBE v.6^50^*.*

PartitionFinder v.2.1.1^51^ was used to reveal the optimal partitioning scheme and nucleotide substitution model, using the Bayesian Information Criterion (BIC). Nucleotide substitution models include K80 + G for the first, HKY + G for the second, and GTR + G for the third codon position. Maximum likelihood (ML) and Bayesian inference (BI) approaches were employed to assess the phylogenetic relationships. Bayesian phylogenetic tree was constructed in MrBayes v.3.2.7a^52^ using two independent runs of four Markov Chain Monte Carlo (MCMC), ran simultaneously for 10 million generations and sampling every 1,000 generations. The first 25% of the sampled trees and estimated parameters were discarded as burn- in. The effective sample size (ESS) value was checked to be above 200 in Tracer v.1.7.1^53^. The consensus phylogenetic tree was then edited in FigTree v.1.4.4 (http://tree.bio.ed.ac.uk/software/figtree/).

Additionally, maximum likelihood analysis was performed in IQ-TREE v.1.6.12^54^ for 10,000 bootstrap steps. Two methods for species delimitation were then used to identify the specific boundaries in the species. These approaches were implemented on online servers: the Poisson Tree Process model (PTP, https://species.h-its.org/ptp/) and the General Mixed Yule Coalescent method (GMYC, https://species.h-its.org/gmyc/).

A Median-Joining (MJ) network analysis was implemented in PopArt v.1.735^55^ to clarify the phylogenetic relationships among haplotypes. Pairwise F_st_ and an analysis of molecular variance (AMOVA) were performed in Arlequin v.3.5.2.2^56^ with 10,000 permutations to estimate the level of genetic structure among clades and subclades in the contact zone.

### Molecular clock analysis

Divergence times of the lineages were estimated using a relaxed lognormal clock model in Beast v. 2.6.4^57^. Two calibration dates of 7.6 and 9 Mya were respectively applied for inferring the minimum ages of the lineages. The first one consisted of *T. kleinmanni* plus *T. marginata* and the second was located between *T. hermanni* and the first calibration node^10^ (the two red circles on the phylogenetic tree, Fig. 2). The best fitting nucleotide substitution model was set for each codon position separately based on the optimal partitioning scheme as mentioned above. The speciation Yule Process (YP) was selected as a tree prior. The MCMC analyses were run for 10 million generations with sampling each 1000 generations and convergence diagnostics were assessed using Tracer v.1.7.1^53^.

### Demographic analysis and neutrality test

Mismatch distributions were estimated separately for *T. g. zarudnyi* and* T. g. buxtoni* to test if their frequency graph shows a chaotic/multimodal pattern characteristic for populations in demographic equilibrium, or an unimodal profile which is found in populations that experienced recent geographic expansion. The test was performed under the null hypothesis that the observed data fit the sudden expansion model, using a generalized least-square approach with 1000 bootstrap replicates. Other statistics for analyzing population expansions or declines were also calculated, i.e., Fu’s FS^58^, Tajima’s D^59^ and Ramos-Onsins and Rozas’s R2^60^ using DnaSP 5.10.1^49^. The negative value of these statistics is interpreted as indicating recent demographic expansion.

### Traditional morphometrics

Body measurements (~ 40 measurements) on different scales of carapace and plastron were performed, using calipers with an accuracy of 0.02 mm. Measured carapace scales consisted of nuchal, five vertebral, and supracaudal scales in the middle, and eight pleural scales on both sides of the vertebral. In plastrons, each scale is divided into two parts by a suture, and the scutes of the plastron include gular, humeral, pectoral, abdominal, femoral and anal.

Traditional morphometric measurements were performed following the protocol of Türkozan et al.^18^: straight carapace length (SCL), from the outermost projection of the cervical scale to the outermost projection of the posteriors marginal; plastron length (PL), from the outermost projection of the gulars to the posterior end of the anal; median carapace width (CW), at the center of the carapace; maximum carapace width (MCW), at posterior marginal 7–9; carapace height (CH), the vertical measurement between the most dorsal point of carapace and the most ventral point of plastron; length of bridge (LB), between axillary and inguinal scales; maximum (not midline) gular scale length (MGSL); maximum (combined left plus right) gular scale width (MGSW); maximum humeral scale width (CHSW); maximum pectoral scale width (CPSW); maximum abdominal scale width (CAbSW); maximum femoral scale suture length width (CFSW); maximum anal scale width (CSAW); gular suture length (GSL), humeral suture length (HSL); pectoral suture length (PSL); abdominal suture length (AbSL); femoral suture length (FSL); anal suture length (ASL); nuchal length (NL), straight-line measurement from the anterior edge of cervical scale to posterior end; nuchal width (NW), the width of cervical at the posterior end; maximum width of first vertebral scale (VW1); maximum width of second vertebral scale (VW2); maximum width of third vertebral scale (VW3); maximum width of fourth vertebral scale (VW4); maximum width of fifth vertebral scale (VW5); maximum median length of first vertebral scale (VL1); maximum median length of second vertebral scale (VL2); maximum median length of third vertebral scale (VL3); maximum median length of fourth vertebral scale (VL4); maximum median length of fifth vertebral scale (VL5); maximum dorsal width of supracaudal scale (DSW); maximum ventral width of supracaudal (VSW); maximum median length of supracaudal length (SL); first costal scale along length, as the minimum straight line distance between the anteriormost and posteriormost contact points with adjacent (normally first and fifth) marginal scales (CL1); length of second costal scale along the marginal (CL2); length of third costal scale along the marginal (CL3); length of fourth costal scale along the marginal (CL4); and maximum inner height of anterior shell opening parallel to median axis (IHASO).

Since the morphological features may change with increasing body size, the effect of dimensions must be reduced and differences between the groups should be due to the differences in body shape and not the relative size. Thus, standardizing morphometric data is required to reduce the changes caused by allometric growth^61^. In order to eliminate the effect of size, morphometric data were standardized prior to performing analyses, using the following formula.

$$\begin{aligned} & {\text{M}}\left( {\text{t}} \right) = {\text{M}}\left( {\text{o}} \right)\left( {{\text{L}}/{\text{L}}\left( 0 \right)} \right)^{{\text{b}}} \\ & {\text{M}}\left( {\text{t}} \right):{\text{Standardized}}\;{\text{values}}\;{\text{of}}\;{\text{variables}} \\ & {\text{M}}\left( {\text{o}} \right):{\text{The}}\;{\text{value}}\;{\text{of}}\;{\text{the}}\;{\text{observed}}\;{\text{variables}} \\ & {\text{L}}:{\text{Average}}\;{\text{standard}}\;{\text{value}}\;{\text{for}}\;{\text{all}}\;{\text{samples}}\;{\text{in}}\;{\text{different}}\;{\text{areas}} \\ & {\text{L}}\left( 0 \right):{\text{Standard}}\;{\text{value}}\;{\text{of}}\;{\text{each}}\;{\text{sample}}\left( {{\text{SCL}}} \right) \\ & {\text{b}}:{\text{Regression}}\;{\text{coefficient}}\;{\text{between}}\;{\text{log}}\;{\text{M}}\left( {\text{o}} \right)\;{\text{and}}\;{\text{log}}\;{\text{L}}\left( {\text{o}} \right)\;{\text{for}}\;{\text{each}}\;{\text{area}} \\ \end{aligned}$$

A cluster analysis was performed to find the optimal number of morphological populations. The sampling localities were grouped into four areas (representative of different landscapes and climatic conditions), including Central part of Zagros Eco-Region (CZ), Southern part of Zagros Eco-Region (SZ), Plain areas of Irano-Turanian Eco-Region (P.I-T) and Mountainous areas of Irano-Turanian Eco-Region (M.I-T). We also recorded the most significant factors shaping the distribution pattern of the individuals, i.e., annual precipitation and temperature seasonality of each area^7,62^ to assess the differences, using one-way analysis of variance (ANOVA) (Table S2). Inter-group variance was evaluated using canonical variate analysis (CVA). Furthermore, a discriminant analysis was performed to classify individuals into known populations. All analyses were performed, using the software PAST^63^ and MINITAB 18^64^.

### Geometric morphometrics

Tortoises were labeled and photographed alive without anesthesia, using a Canon 350 D digital camera (8 MP). Photographic conditions such as camera settings, magnification, focus, lens size, camera distance from the sample surface and backlight were consistent for all individuals and images were taken from ventral and dorsal views. The camera was placed parallel to the ground plane, as such that the focal axis of the camera was parallel to the horizontal plane of reference and centered on the plastron and carapace.

**Figure 11:**
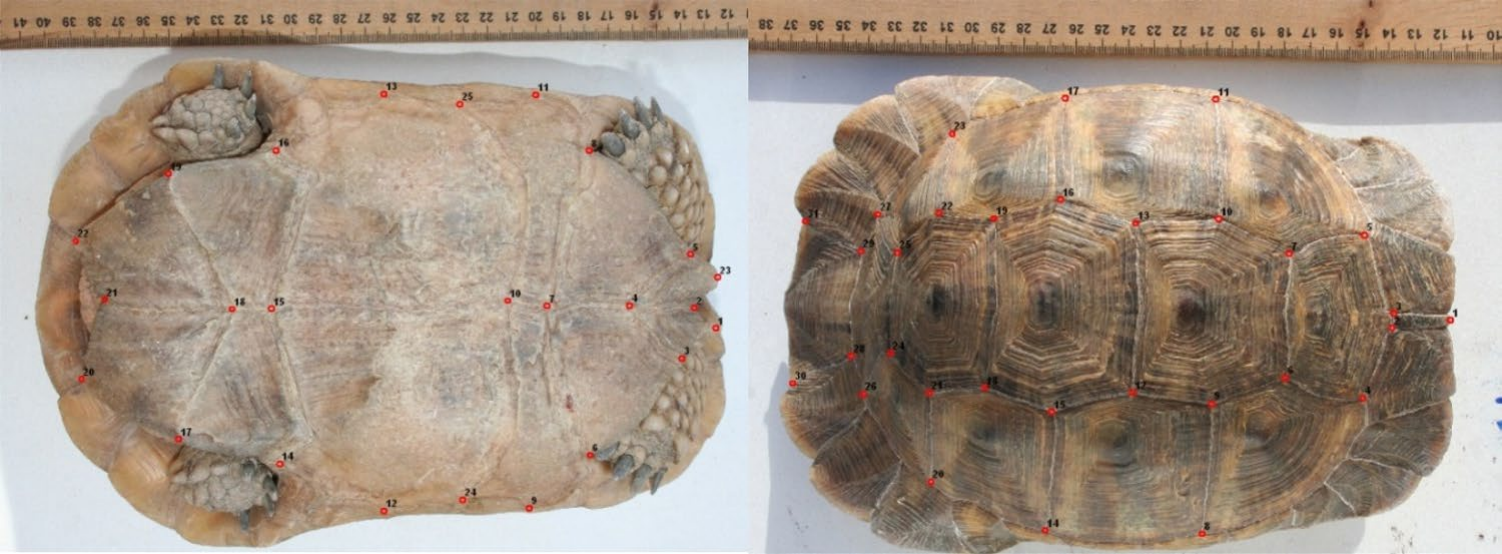
The defined landmarks for extracting the shape data of T. graeca in Central Iran.

On each image, 2D coordinates of fixed landmarks were digitized by the same person, using TPS DIG v. 2.31^65^. In total, 31 and 25 two-dimensional (2D) landmarks were digitized on carapace and plastron, respectively (Fig. 11). Landmarks were digitized on the intersections of dermal scales. The distribution of landmarks covers nuchal, vertebral, pleural, and supracaudal scales of the carapace and twelve left and right scales of plastron (gular, humeral, pectoral, abdominal, femoral and anal). To minimize observer-induced errors, one person digitized the landmarks. Landmarks were, then, analyzed using generalized Procrustes analysis (GPA)^66,67^, which mathematically removes the effects of non-shape variation and normalizes shape data at equal scale, allowing for an accurate comparison of shapes regardless of their position, orientation and isometric size. To evaluate the differences among populations, CVA was performed on the shape data. Finally, cluster analysis (CA) was performed to group the studied populations. All analyses were conducted using PAST^63^ and MorphoJ^68^.

## Supplementary Information

Supplementary Information.

## Data Availability

DNA sequences have been deposited in GenBank (https://www.ncbi.nlm.nih.gov/genbank/) under the accession no. ON887288-ON887307.
