# Supplementary material for: Phylogeographic and phenotypic divergence between two subspecies of Testudo graeca (T. g. buxtoni and T. g. zarudnyi) across their contact zone in Iran
Source: Sci Rep. 2022 Aug 9;12:13579. doi: 10.1038/s41598-022-17926-1 (PMC9363490; doi:10.1038/s41598-022-17926-1)
Supplement: Supplementary file 1 — Supplementary Information. [file 41598_2022_17926_MOESM1_ESM.docx]

**Table S1.** List of *Testudo graeca* samples used in this study

| No | Subspecies | Hap | Sample ID | country | location | reference | Acc. No |
| --- | --- | --- | --- | --- | --- | --- | --- |
| 1 | *T. g. buxtoni* | 1 | NR1 | Iran | Falard | This study | ON887288 |
| 2 | *T. g. buxtoni* | 2 | NR2 | Iran | Ilan Dareh-Semirom | This study | ON887289 |
| 3 | *T. g. buxtoni* | 1 | NR3 | Iran | Ilan Dareh-Semirom | This study | ON887290 |
| 4 | *T. g. zarudnyi* | 3 | NR4 | Iran | Kolah Qazi National Park | This study | ON887291 |
| 5 | *T. g. zarudnyi* | 4 | NR5 | Iran | Ghuri; Neyriz | This study | ON887292 |
| 6 | *T. g. zarudnyi* | 3 | NR6 | Iran | Pariz; Sirjan | This study | ON887293 |
| 7 | *T. g. zarudnyi* | 5 | NR7 | Iran | Rafsanjan | This study | ON887294 |
| 8 | *T. g. zarudnyi* | 5 | NR8 | Iran | Rafsanjan | This study | ON887295 |
| 9 | *T. g. zarudnyi* | 5 | NR9 | Iran | Kerman | This study | ON887296 |
| 10 | *T. g. zarudnyi* | 3 | NR10 | Iran | Marvar; Meybod | This study | ON887297 |
| 11 | *T. g. zarudnyi* | 3 | NR11 | Iran | Abadeh; Fars | This study | ON887298 |
| 12 | *T. g. buxtoni* | 6 | NR12 | Iran | Fasa; Fars | This study | ON887299 |
| 13 | *T. g. zarudnyi* | 3 | NR13 | Iran | Bamou National Park; Shiraz | This study | ON887300 |
| 14 | *T. g. buxtoni* | 7 | NR14 | Iran | Dalankouh; Fereidan | This study | ON887301 |
| 15 | *T. g. buxtoni* | 8 | NR15 | Iran | Bamou National Park; Shiraz | This study | ON887302 |
| 16 | *T. g. zarudnyi* | 5 | NR16 | Iran | Kouh-e-Bafgh; Yazd | This study | ON887303 |
| 17 | *T. g. zarudnyi* | 3 | NR17 | Iran | Mehriz; Yazd | This study | ON887304 |
| 18 | *T. g. zarudnyi* | 3 | NR18 | Iran | Hassan Abad; Yazd | This study | ON887305 |
| 19 | *T. g. zarudnyi* | 3 | NR19 | Iran | Ardakan; Yazd | This study | ON887306 |
| 20 | *T. g. zarudnyi* | 5 | NR20 | Iran | Abbas Abad Wildlife Refuge- Chupanan | This study | ON887307 |
| 21 | *T. g. armenaica* | 9 | FK015 | Turkey | Aralık | Turkozan,et al. 2017 | MF494408 |
| 22 | *T. g. armenaica* | 9 | FK016 | Turkey | Aralık | Turkozan,et al. 2017 | MF494407 |
| 23 | *T. g. ibera* | 10 | MB193 | Turkey | Zonguldak | Turkozan,et al. 2017 | MF494406 |
| 24 | *T. g. armenaica* | 12 | MB28 | Turkey | Aralık | Turkozan,et al. 2017 | MF494403 |
| 25 | *T. g. armenaica* | 12 | MB5 | Turkey | Aralık | Turkozan,et al. 2017 | MF494402 |
| 26 | *T. g. armenaica* | 12 | MB27 | Turkey | Aralık | Turkozan,et al. 2017 | MF494401 |
| 27 | *T. g. buxtoni* | 13 | MB20 | Turkey | Yüksekova | Turkozan,et al. 2017 | MF494400 |
| 28 | *T. g. buxtoni* | 14 | MB19 | Turkey | Şırnak | Turkozan,et al. 2017 | MF494399 |
| 29 | *T. g. buxtoni* | 14 | MB18 | Turkey | Şırnak | Turkozan,et al. 2017 | MF494398 |
| 30 | *T. g. buxtoni* | 15 | MB40 | Turkey | Yüksekova | Turkozan,et al. 2017 | MF494397 |
| 31 | *T. g. buxtoni* | 16 | MB24 | Turkey | Başkale | Turkozan,et al. 2017 | MF494396 |
| 32 | *T. g. buxtoni* | 17 | MB23 | Turkey | Başkale | Turkozan,et al. 2017 | MF494395 |
| 33 | *T. g. buxtoni* | 18 | MB22 | Turkey | Başkale | Turkozan,et al. 2017 | MF494394 |
| 34 | *T. g. buxtoni* | 19 | MB21 | Turkey | Yüksekova | Turkozan,et al. 2017 | MF494393 |
| 35 | *T. g. buxtoni* | 20 | MB17 | Turkey | Şırnak | Turkozan,et al. 2017 | MF494392 |
| 36 | *T. g. buxtoni* | 14 | MB124 | Turkey | Şırnak | Turkozan,et al. 2017 | MF494391 |
| 37 | *T. g. buxtoni* | 13 | MB123 | Turkey | Yüksekova | Turkozan,et al. 2017 | MF494390 |
| 38 | *T. g. ibera* | 10 | FKAYV | Turkey | Ayvalık | Turkozan,et al. 2017 | MF494389 |
| 39 | *T. g. ibera* | 10 | FK38CRM | Turkey | Çorum | Turkozan,et al. 2017 | MF494388 |
| 40 | *T. g. ibera* | 10 | FK37CRM | Turkey | Çorum | Turkozan,et al. 2017 | MF494387 |
| 41 | *T. g. ibera* | 10 | FK36AMSY | Turkey | Amasya | Turkozan,et al. 2017 | MF494386 |
| 42 | *T. g. ibera* | 21 | FK34 | Turkey | Çamlıbel, Tokat | Turkozan,et al. 2017 | MF494385 |
| 43 | *T. g. ibera* | 21 | FK32SVS | Turkey | Sivas | Turkozan,et al. 2017 | MF494384 |
| 44 | *T. g. ibera* | 21 | FK30NVSH | Turkey | Nevşehir | Turkozan,et al. 2017 | MF494383 |
| 45 | *T. g. ibera* | 21 | FK29 | Turkey | Nevşehir | Turkozan,et al. 2017 | MF494382 |
| 46 | *T. g. ibera* | 21 | FK28 | Turkey | Nevşehir | Turkozan,et al. 2017 | MF494381 |
| 47 | *T. g. ibera* | 22 | FK27 | Turkey | Ürgüp_Avanos road | Turkozan,et al. 2017 | MF494380 |
| 48 | *T. g. ibera* | 21 | FK26 | Turkey | Ürgüp_Avanos road | Turkozan,et al. 2017 | MF494379 |
| 49 | *T. g. ibera* | 22 | MB908 | Turkey | Tavas | Turkozan,et al. 2017 | MF494378 |
| 50 | *T. g. ibera* | 22 | MB907 | Turkey | Tavas | Turkozan,et al. 2017 | MF494377 |
| 51 | *T. g. ibera* | 22 | MB906 | Turkey | Acıpayam | Turkozan,et al. 2017 | MF494376 |
| 52 | *T. g. ibera* | 10 | MB905 | Turkey | Acıpayam | Turkozan,et al. 2017 | MF494375 |
| 53 | *T. g. ibera* | 10 | MB904 | Turkey | Harmanlı_Burdur road | Turkozan,et al. 2017 | MF494374 |
| 54 | *T. g. ibera* | 10 | MB902 | Turkey | İşcehisar | Turkozan,et al. 2017 | MF494373 |
| 55 | *T. g. ibera* | 22 | MB901 | Turkey | İşcehisar | Turkozan,et al. 2017 | MF494372 |
| 56 | *T. g. ibera* | 22 | MB64 | Turkey | Afyon_Antalya road | Turkozan,et al. 2017 | MF494371 |
| 57 | *T. g. ibera* | 10 | MB61 | Turkey | Safranbolu | Turkozan,et al. 2017 | MF494370 |
| 58 | *T. g. ibera* | 10 | MB60 | Turkey | Mengen | Turkozan,et al. 2017 | MF494369 |
| 59 | *T. g. ibera* | 10 | MB58 | Turkey | Tavas | Turkozan,et al. 2017 | MF494368 |
| 60 | *T. g. ibera* | 10 | MB57 | Turkey | Çay_Afyon road | Turkozan,et al. 2017 | MF494367 |
| 61 | *T. g. ibera* | 10 | MB513 | Turkey | Gölcük | Turkozan,et al. 2017 | MF494366 |
| 62 | *T. g. ibera* | 10 | MB512 | Turkey | Gölcük | Turkozan,et al. 2017 | MF494365 |
| 63 | *T. g. ibera* | 10 | MB511 | Turkey | Bilecik | Turkozan,et al. 2017 | MF494364 |
| 64 | *T. g. ibera* | 10 | MB510 | Turkey | Bilecik | Turkozan,et al. 2017 | MF494363 |
| 65 | *T. g. ibera* | 10 | MB51 | Turkey | Amasra | Turkozan,et al. 2017 | MF494362 |
| 66 | *T. g. ibera* | 10 | MB509 | Turkey | Bilecik | Turkozan,et al. 2017 | MF494361 |
| 67 | *T. g. ibera* | 10 | MB508 | Turkey | Bursa | Turkozan,et al. 2017 | MF494360 |
| 68 | *T. g. ibera* | 10 | MB507 | Turkey | Bursa | Turkozan,et al. 2017 | MF494359 |
| 69 | *T. g. ibera* | 10 | MB506 | Turkey | Bursa | Turkozan,et al. 2017 | MF494358 |
| 70 | *T. g. ibera* | 10 | MB505 | Turkey | Bandırma | Turkozan,et al. 2017 | MF494357 |
| 71 | *T. g. ibera* | 10 | MB504 | Turkey | Balıkesir | Turkozan,et al. 2017 | MF494356 |
| 72 | *T. g. ibera* | 10 | MB503 | Turkey | Balıkesir | Turkozan,et al. 2017 | MF494355 |
| 73 | *T. g. ibera* | 10 | MB502 | Turkey | Truva | Turkozan,et al. 2017 | MF494354 |
| 74 | *T. g. ibera* | 10 | MB501 | Turkey | Truva | Turkozan,et al. 2017 | MF494353 |
| 75 | *T. g. ibera* | 10 | MB49 | Turkey | Amasra | Turkozan,et al. 2017 | MF494352 |
| 76 | *T. g. ibera* | 22 | MB416 | Turkey | Kütahya_Afyon road | Turkozan,et al. 2017 | MF494351 |
| 77 | *T. g. ibera* | 22 | MB415 | Turkey | Şile | Turkozan,et al. 2017 | MF494350 |
| 78 | *T. g. ibera* | 22 | MB414 | Turkey | Sapanca | Turkozan,et al. 2017 | MF494349 |
| 79 | *T. g. ibera* | 22 | MB413 | Turkey | Sapanca | Turkozan,et al. 2017 | MF494348 |
| 80 | *T. g. ibera* | 22 | MB412 | Turkey | Gölcük | Turkozan,et al. 2017 | MF494347 |
| 81 | *T. g. ibera* | 22 | MB411 | Turkey | Şarköy_Evreşe road | Turkozan,et al. 2017 | MF494346 |
| 82 | *T. g. ibera* | 22 | MB410 | Turkey | Şarköy | Turkozan,et al. 2017 | MF494345 |
| 83 | *T. g. ibera* | 22 | MB409 | Turkey | Bahçeköy | Turkozan,et al. 2017 | MF494344 |
| 84 | *T. g. ibera* | 22 | MB408 | Turkey | Çakıllı | Turkozan,et al. 2017 | MF494343 |
| 85 | *T. g. ibera* | 22 | MB407 | Turkey | Vize | Turkozan,et al. 2017 | MF494342 |
| 86 | *T. g. ibera* | 22 | MB406 | Turkey | Pınarhisar | Turkozan,et al. 2017 | MF494341 |
| 87 | *T. g. ibera* | 22 | MB404 | Turkey | Hanlıyenice | Turkozan,et al. 2017 | MF494340 |
| 88 | *T. g. ibera* | 22 | MB403 | Turkey | Karaağaç | Turkozan,et al. 2017 | MF494339 |
| 89 | *T. g. ibera* | 22 | MB402 | Turkey | Karaağaç | Turkozan,et al. 2017 | MF494338 |
| 90 | *T. g. ibera* | 22 | MB401 | Turkey | Gölmarmara | Turkozan,et al. 2017 | MF494337 |
| 91 | *T. g. ibera* | 22 | MB400 | Turkey | Gölmarmara | Turkozan,et al. 2017 | MF494336 |
| 92 | *T. g. ibera* | 10 | MB37 | Turkey | Taşköprü | Turkozan,et al. 2017 | MF494335 |
| 93 | *T. g. ibera* | 22 | MB303 | Turkey | Nallıhan | Turkozan,et al. 2017 | MF494334 |
| 94 | *T. g. ibera* | 22 | MB302 | Turkey | Nallıhan | Turkozan,et al. 2017 | MF494333 |
| 95 | *T. g. ibera* | 22 | MB301 | Turkey | Cihanbeyli | Turkozan,et al. 2017 | MF494332 |
| 96 | *T. g. ibera* | 10 | MB300 | Turkey | Araç_Kastamonu road | Turkozan,et al. 2017 | MF494331 |
| 97 | *T. g. ibera* | 23 | MB204 | Turkey | Karapınar | Turkozan,et al. 2017 | MF494330 |
| 98 | *T. g. ibera* | 23 | MB201 | Turkey | Karapınar | Turkozan,et al. 2017 | MF494329 |
| 99 | *T. g. ibera* | 23 | MB200 | Turkey | Karapınar | Turkozan,et al. 2017 | MF494328 |
| 100 | *T. g. ibera* | 10 | MB192 | Turkey | Zonguldak | Turkozan,et al. 2017 | MF494327 |
| 101 | *T. g. ibera* | 10 | MB191 | Turkey | Zonguldak | Turkozan,et al. 2017 | MF494326 |
| 102 | *T. g. ibera* | 10 | MB190 | Turkey | Safranbolu | Turkozan,et al. 2017 | MF494325 |
| 103 | *T. g. ibera* | 10 | MB179 | Turkey | Samsun | Turkozan,et al. 2017 | MF494324 |
| 104 | *T. g. ibera* | 10 | MB178 | Turkey | Sinop | Turkozan,et al. 2017 | MF494323 |
| 105 | *T. g. ibera* | 10 | MB177 | Turkey | Sinop | Turkozan,et al. 2017 | MF494322 |
| 106 | *T. g. ibera* | 10 | MB176 | Turkey | Tosya | Turkozan,et al. 2017 | MF494321 |
| 107 | *T. g. ibera* | 10 | MB175 | Turkey | Samsun | Turkozan,et al. 2017 | MF494320 |
| 108 | *T. g. ibera* | 10 | MB174 | Turkey | Samsun | Turkozan,et al. 2017 | MF494319 |
| 109 | *T. g. ibera* | 10 | MB173 | Turkey | Amasya | Turkozan,et al. 2017 | MF494318 |
| 110 | *T. g. ibera* | 10 | MB172 | Turkey | Amasya | Turkozan,et al. 2017 | MF494317 |
| 111 | *T. g. ibera* | 10 | MB171 | Turkey | Amasya | Turkozan,et al. 2017 | MF494316 |
| 112 | *T. g. terrestris* | 24 | MB130 | Turkey | Maraş | Turkozan,et al. 2017 | MF494315 |
| 113 | *T. g. ibera* | 22 | MB903 | Turkey | Çay | Turkozan,et al. 2017 | MF494314 |
| 114 | *T. g. ibera* | 10 | MB54 | Turkey | Sandıklı_Dinar road | Turkozan,et al. 2017 | MF494313 |
| 115 | *T. g. ibera* | 25 | MB500 | Turkey | Şarköy_Evreşe road | Turkozan,et al. 2017 | MF494312 |
| 116 | *T. g. ibera* | 10 | MB48 | Turkey | İnebolu | Turkozan,et al. 2017 | MF494311 |
| 117 | *T. g. ibera* | 22 | MB405 | Turkey | Hanlıyenice | Turkozan,et al. 2017 | MF494310 |
| 118 | *T. g. ibera* | 26 | MB4 | Turkey | Akbelen | Turkozan,et al. 2017 | MF494309 |
| 119 | *T. g. ibera* | 27 | MB203 | Turkey | Karapınar | Turkozan,et al. 2017 | MF494308 |
| 120 | *T. g. ibera* | 23 | MB202 | Turkey | Karapınar | Turkozan,et al. 2017 | MF494307 |
| 121 | *T. g. ibera* | 23 | MB2 | Turkey | Karapınar | Turkozan,et al. 2017 | MF494306 |
| 122 | *T. g. ibera* | 22 | FK3KUT | Turkey | Kütahya | Turkozan,et al. 2017 | MF494305 |
| 123 | *T. g. ibera* | 26 | FK35 | Turkey | Suşehri, Sivas | Turkozan,et al. 2017 | MF494304 |
| 124 | *T. g. ibera* | 21 | FK31 | Turkey | Sivas | Turkozan,et al. 2017 | MF494303 |
| 125 | *T. g. ibera* | 28 | FK2ERZ | Turkey | Kemaliye | Turkozan,et al. 2017 | MF494302 |
| 126 | *T. g. ibera* | 21 | FK25 | Turkey | Ürgüp_Avanos road | Turkozan,et al. 2017 | MF494301 |
| 127 | *T. g. ibera* | 22 | FK24 | Turkey | Yozgat | Turkozan,et al. 2017 | MF494300 |
| 128 | *T. g. ibera* | 10 | FK23YZGT | Turkey | Yozgat | Turkozan,et al. 2017 | MF494299 |
| 129 | *T. g. ibera* | 10 | FK33SVS | Turkey | Sivas | Turkozan,et al. 2017 | MF494298 |
| 130 | *T. g. ibera* | 10 | FK4AYV | Turkey | Ayvalık | Turkozan,et al. 2017 | MF494297 |
| 131 | *T. g. terrestris* | 24 | FK22 | Turkey | Maraş | Turkozan,et al. 2017 | MF494296 |
| 132 | *T. g. terrestris* | 24 | FK20KLS | Turkey | Kilis | Turkozan,et al. 2017 | MF494295 |
| 133 | *T. g. terrestris* | 24 | FK19 | Turkey | Dörtyol | Turkozan,et al. 2017 | MF494294 |
| 134 | *T. g. terrestris* | 29 | FK14CYH | Turkey | İncirli | Turkozan,et al. 2017 | MF494293 |
| 135 | *T. g. terrestris* | 29 | FK10ADN | Turkey | Adana | Turkozan,et al. 2017 | MF494292 |
| 136 | *T. g. terrestris* | 29 | MB900 | Turkey | Eğirdir | Turkozan,et al. 2017 | MF494291 |
| 137 | *T. g. terrestris* | 30 | MB9 | Turkey | Batman | Turkozan,et al. 2017 | MF494290 |
| 138 | *T. g. terrestris* | 31 | MB805 | Turkey | Hadim | Turkozan,et al. 2017 | MF494289 |
| 139 | *T. g. terrestris* | 29 | MB802 | Turkey | Gazipaşa-Anamur road | Turkozan,et al. 2017 | MF494288 |
| 140 | *T. g. terrestris* | 29 | MB800 | Turkey | Bozyazı-Gülnar road | Turkozan,et al. 2017 | MF494287 |
| 141 | *T. g. terrestris* | 32 | MB8 | Turkey | Diyarbakır | Turkozan,et al. 2017 | MF494286 |
| 142 | *T. g. terrestris* | 29 | MB709 | Turkey | Demre | Turkozan,et al. 2017 | MF494285 |
| 143 | *T. g. terrestris* | 29 | MB708 | Turkey | Kalkan-Kaş road | Turkozan,et al. 2017 | MF494284 |
| 144 | *T. g. terrestris* | 29 | MB707 | Turkey | Letoon | Turkozan,et al. 2017 | MF494283 |
| 145 | *T. g. terrestris* | 29 | MB706 | Turkey | Letoon | Turkozan,et al. 2017 | MF494282 |
| 146 | *T. g. terrestris* | 29 | MB705 | Turkey | Letoon | Turkozan,et al. 2017 | MF494281 |
| 147 | *T. g. terrestris* | 29 | MB704 | Turkey | Günlüklü | Turkozan,et al. 2017 | MF494280 |
| 148 | *T. g. terrestris* | 29 | MB703 | Turkey | Günlüklü | Turkozan,et al. 2017 | MF494279 |
| 149 | *T. g. terrestris* | 24 | MB66 | Turkey | unknown exact origin | Turkozan,et al. 2017 | MF494278 |
| 150 | *T. g. terrestris* | 33 | MB63 | Turkey | Diyarbakır | Turkozan,et al. 2017 | MF494277 |
| 151 | *T. g. terrestris* | 29 | MB59 | Turkey | Eğirdir | Turkozan,et al. 2017 | MF494276 |
| 152 | *T. g. terrestris* | 29 | MB55 | Turkey | Perge | Turkozan,et al. 2017 | MF494275 |
| 153 | *T. g. terrestris* | 29 | MB53 | Turkey | Silifke-Muş road | Turkozan,et al. 2017 | MF494274 |
| 154 | *T. g. terrestris* | 34 | MB43 | Turkey | Mercimekkale | Turkozan,et al. 2017 | MF494273 |
| 155 | *T. g. terrestris* | 35 | MB42 | Turkey | Genç, Bingöl | Turkozan,et al. 2017 | MF494272 |
| 156 | *T. g. terrestris* | 24 | MB39 | Turkey | Kilis-Hassa road | Turkozan,et al. 2017 | MF494271 |
| 157 | *T. g. terrestris* | 24 | MB36 | Turkey | Malatya | Turkozan,et al. 2017 | MF494270 |
| 158 | *T. g. terrestris* | 24 | MB33 | Turkey | Gaziantep | Turkozan,et al. 2017 | MF494269 |
| 159 | *T. g. terrestris* | 24 | MB32 | Turkey | Gaziantep | Turkozan,et al. 2017 | MF494268 |
| 160 | *T. g. terrestris* | 29 | MB31 | Turkey | Isparta_Ağlasun road | Turkozan,et al. 2017 | MF494267 |
| 161 | *T. g. terrestris* | 35 | MB29 | Turkey | Genç, Bingöl | Turkozan,et al. 2017 | MF494266 |
| 162 | *T. g. terrestris* | 36 | MB26 | Turkey | Erciş | Turkozan,et al. 2017 | MF494265 |
| 163 | *T. g. terrestris* | 30 | MB15 | Turkey | Yelkenli | Turkozan,et al. 2017 | MF494264 |
| 164 | *T. g. terrestris* | 37 | MB142 | Turkey | İncirli | Turkozan,et al. 2017 | MF494263 |
| 165 | *T. g. terrestris* | 37 | MB141 | Turkey | Dörtyol | Turkozan,et al. 2017 | MF494262 |
| 166 | *T. g. terrestris* | 37 | MB140 | Turkey | Dörtyol | Turkozan,et al. 2017 | MF494261 |
| 167 | *T. g. terrestris* | 34 | MB122 | Turkey | Mercimekkale | Turkozan,et al. 2017 | MF494260 |
| 168 | *T. g. terrestris* | 29 | MB143 | Turkey | İncirli | Turkozan,et al. 2017 | MF494259 |
| 169 | *T. g. terrestris* | 38 | MB62 | Turkey | Elazığ | Turkozan,et al. 2017 | MF494258 |
| 170 | *T. g. terrestris* | 29 | MB809 | Turkey | Kumluca_Kemer road | Turkozan,et al. 2017 | MF494257 |
| 171 | *T. g. terrestris* | 39 | MB808 | Turkey | Kızılot_Akseki road | Turkozan,et al. 2017 | MF494256 |
| 172 | *T. g. terrestris* | 31 | MB804 | Turkey | Hadim | Turkozan,et al. 2017 | MF494255 |
| 173 | *T. g. terrestris* | 39 | MB710 | Turkey | Manavgat_Akseki road | Turkozan,et al. 2017 | MF494254 |
| 174 | *T. g. terrestris* | 29 | MB702 | Turkey | Günlüklü | Turkozan,et al. 2017 | MF494253 |
| 175 | *T. g. terrestris* | 29 | MB701 | Turkey | Kızılağaç | Turkozan,et al. 2017 | MF494252 |
| 176 | *T. g. terrestris* | 29 | MB700 | Turkey | Isparta_Ağlasun road | Turkozan,et al. 2017 | MF494251 |
| 177 | *T. g. terrestris* | 40 | MB7 | Turkey | Diyarbakır | Turkozan,et al. 2017 | MF494250 |
| 178 | *T. g. terrestris* | 41 | MB6 | Turkey | Antakya | Turkozan,et al. 2017 | MF494249 |
| 179 | *T. g. terrestris* | 29 | MB52 | Turkey | Gazipaşa-Anamur road | Turkozan,et al. 2017 | MF494248 |
| 180 | *T. g. terrestris* | 29 | MB50 | Turkey | Karapınar | Turkozan,et al. 2017 | MF494247 |
| 181 | *T. g. terrestris* | 35 | MB45 | Turkey | Şanlıurfa | Turkozan,et al. 2017 | MF494246 |
| 182 | *T. g. terrestris* | 24 | MB35 | Turkey | Adıyaman | Turkozan,et al. 2017 | MF494245 |
| 183 | *T. g. terrestris* | 35 | MB30 | Turkey | Genç, Bingöl | Turkozan,et al. 2017 | MF494244 |
| 184 | *T. g. terrestris* | 42 | MB3 | Turkey | Diyarbakır | Turkozan,et al. 2017 | MF494243 |
| 185 | *T. g. terrestris* | 36 | MB25 | Turkey | Erciş | Turkozan,et al. 2017 | MF494242 |
| 186 | *T. g. terrestris* | 43 | MB16 | Turkey | Yelkenli | Turkozan,et al. 2017 | MF494241 |
| 187 | *T. g. terrestris* | 44 | MB150 | Turkey | Çullu | Turkozan,et al. 2017 | MF494240 |
| 188 | *T. g. terrestris* | 30 | MB14 | Turkey | Yelkenli | Turkozan,et al. 2017 | MF494239 |
| 189 | *T. g. terrestris* | 24 | MB131 | Turkey | Antakya | Turkozan,et al. 2017 | MF494238 |
| 190 | *T. g. terrestris* | 45 | MB13 | Turkey | Eruh | Turkozan,et al. 2017 | MF494237 |
| 191 | *T. g. terrestris* | 32 | MB126 | Turkey | Mardin | Turkozan,et al. 2017 | MF494236 |
| 192 | *T. g. terrestris* | 32 | MB125 | Turkey | Mardin | Turkozan,et al. 2017 | MF494235 |
| 193 | *T. g. terrestris* | 34 | MB121 | Turkey | Mercimekkale | Turkozan,et al. 2017 | MF494234 |
| 194 | *T. g. terrestris* | 35 | MB120 | Turkey | Genç, Bingöl | Turkozan,et al. 2017 | MF494233 |
| 195 | *T. g. terrestris* | 46 | MB12 | Turkey | Batman | Turkozan,et al. 2017 | MF494232 |
| 196 | *T. g. terrestris* | 47 | MB11 | Turkey | Batman | Turkozan,et al. 2017 | MF494231 |
| 197 | *T. g. terrestris* | 48 | MB10 | Turkey | Batman | Turkozan,et al. 2017 | MF494230 |
| 198 | *T. g. terrestris* | 30 | FK9VAN | Turkey | Yelkenli | Turkozan,et al. 2017 | MF494229 |
| 199 | *T. g. terrestris* | 49 | FK8VAN | Turkey | Yelkenli | Turkozan,et al. 2017 | MF494228 |
| 200 | *T. g. terrestris* | 33 | FK7DBKR | Turkey | Diyarbakır | Turkozan,et al. 2017 | MF494227 |
| 201 | *T. g. terrestris* | 24 | FK6ISK | Turkey | İskenderun | Turkozan,et al. 2017 | MF494226 |
| 202 | *T. g. terrestris* | 50 | FK5ANT | Turkey | Antakya | Turkozan,et al. 2017 | MF494225 |
| 203 | *T. g. terrestris* | 44 | FK41KDRL | Turkey | Kadirli | Turkozan,et al. 2017 | MF494224 |
| 204 | *T. g. terrestris* | 51 | FK40SAIM | Turkey | Saimbeyli | Turkozan,et al. 2017 | MF494223 |
| 205 | *T. g. terrestris* | 51 | FK39SAIMB | Turkey | Saimbeyli | Turkozan,et al. 2017 | MF494222 |
| 206 | *T. g. terrestris* | 24 | FK21MLTY | Turkey | Malatya | Turkozan,et al. 2017 | MF494221 |
| 207 | *T. g. terrestris* | 52 | FK1ANT | Turkey | Kırıkhan | Turkozan,et al. 2017 | MF494220 |
| 208 | *T. g. terrestris* | 24 | FK18 | Turkey | Antakya | Turkozan,et al. 2017 | MF494219 |
| 209 | *T. g. terrestris* | 24 | FK17 | Turkey | Maraş | Turkozan,et al. 2017 | MF494218 |
| 210 | *T. g. terrestris* | 24 | FK16ANT | Turkey | Dörtyol | Turkozan,et al. 2017 | MF494217 |
| 211 | *T. g. terrestris* | 24 | FK15ANT | Turkey | Dörtyol | Turkozan,et al. 2017 | MF494216 |
| 212 | *T. g. terrestris* | 37 | FK13CYH | Turkey | Ceyhan | Turkozan,et al. 2017 | MF494215 |
| 213 | *T. g. terrestris* | 29 | FK11CYH | Turkey | Ceyhan | Turkozan,et al. 2017 | MF494214 |
| 214 | *T. g. terrestris* | 29 | MB44 | Turkey | İncirli | Turkozan,et al. 2017 | MF494213 |
| 215 | *T. g. terrestris* | 38 | MB41 | Turkey | Elazığ | Turkozan,et al. 2017 | MF494212 |
| 216 | *T. g. terrestris* | 53 | MB1 | Turkey | Malatya | Turkozan,et al. 2017 | MF494211 |
| 217 | *T. g. terrestris* | 39 | MB807 | Turkey | Kızılot_Akseki road | Turkozan,et al. 2017 | MF494210 |
| 218 | *T. g. terrestris* | 29 | MB806 | Turkey | Akseki | Turkozan,et al. 2017 | MF494209 |
| 219 | *T. g. terrestris* | 29 | MB803 | Turkey | Gazipaşa-Anamur road | Turkozan,et al. 2017 | MF494208 |
| 220 | *T. g. graeca* | 54 | M-MO03 | Morocco | Moulouya | Graciá et al., 2013 | HE588138 |
| 221 | *T. g. graeca* | 55 | A-SS08 | Algeria | Saf Saf | Graciá et al., 2013 | HE588137 |
| 222 | *T. g. graeca* | 56 | M-MO07 | Morocco | Moulouya | Graciá et al., 2013 | HE585737 |
| 223 | *T. g. graeca* | 57 | A-AC10 | Algeria | Ain Chorfa | Graciá et al., 2013 | HE585736 |
| 224 | *T. g. graeca* | 58 | A-GU10 | Algeria | Guertofa | Graciá et al., 2013 | HE585735 |
| 225 | *T. g. graeca* | 59 | A-MS09 | Algeria | Messad | Graciá et al., 2013 | HE585734 |
| 226 | *T. g. graeca* | 60 | A-SB01 | Algeria | Sidi M'Hamed Benaouda | Graciá et al., 2013 | HE585733 |
| 227 | *T. g. graeca* | 61 | A-GU11 | Algeria | Guertofa | Graciá et al., 2013 | HE585732 |
| 228 | *T. g. graeca* | 62 | A-SS06 | Algeria | Saf Saf | Graciá et al., 2013 | HE585731 |
| 229 | *T. g. graeca* | 63 | A-SS04 | Algeria | Saf Saf | Graciá et al., 2013 | HE585730 |
| 230 | *T. g. graeca* | 64 | A-SS07 | Algeria | Saf Saf | Graciá et al., 2013 | HE585729 |
| 231 | *T. g. graeca* | 65 | A-ZE01 | Algeria | Zemouri-Leguata | Graciá et al., 2013 | HE585728 |
| 232 | *T. g. graeca* | 66 | A-TH05 | Algeria | Theniet el Had | Graciá et al., 2013 | HE585727 |
| 233 | *T. g. graeca* | 67 | A-MB00 | Algeria | Moudjbara | Graciá et al., 2013 | HE585726 |
| 234 | *T. g. graeca* | 68 | A-AN01 | Algeria | Ain Naga | Graciá et al., 2013 | HE585725 |
| 235 | *T. g. graeca* | 69 | A-AR01 | Algeria | Algiers | Graciá et al., 2013 | HE585724 |
| 236 | *T. g. graeca* | 70 | A-KH04 | Algeria | Zemmora | Graciá et al., 2013 | HE585723 |
| 237 | *T. g. graeca* | 71 | A-GU03 | Algeria | Guertofa | Graciá et al., 2013 | HE585722 |
| 238 | *T. g. graeca* | 72 | A-KH03 | Algeria | Zemmora | Graciá et al., 2013 | HE585721 |
| 239 | *T. g. graeca* | 73 | A-GU01 | Algeria | Guertofa | Graciá et al., 2013 | HE585720 |
| 240 | *T. g. graeca* | 74 | CU06 | Spain | Culebras | Graciá et al., 2013 | HE585719 |
| 241 | *T. g. graeca* | 75 | MA03 | Spain | Marinica | Graciá et al., 2013 | HE585718 |
| 242 | *T. g. graeca* | 76 | A-MS07 | Algeria | Messad | Graciá et al., 2013 | HE585717 |
| 243 | *T. g. graeca* | 77 | A-MB01 | Algeria | Moudjbara | Graciá et al., 2013 | HE585716 |
| 244 | *T. g. graeca* | 78 | A-MS14 | Algeria | Messad | Graciá et al., 2013 | HE585715 |
| 245 | *T. g. graeca* | 79 | A-MS05 | Algeria | Messad | Graciá et al., 2013 | HE585714 |
| 246 | *T. g. graeca* | 80 | M-MO02 | Morocco | Moulouya | Graciá et al., 2013 | HE585713 |
| 247 | *New discovereded lineage* | 81 | 6452 | Libya | unknown exact origin | Graciá et al., 2017 | HE585748 |
| 248 | *T. g. soussensis* | 82 | 5272 | Morocco | near Rabat | Graciá et al., 2017 | HE585747 |
| 249 | *T. g. soussensis* | 83 | 5441 | Morocco | Taroudannt | Graciá et al., 2017 | HE585746 |
| 250 | *T. g. marokkensis* | 84 | 5452 | Morocco | Oulmes | Graciá et al., 2017 | HE585745 |
| 251 | *T. g. marokkensis* | 85 | M-KA05 | Morocco | Sidi Redouane | Graciá et al., 2017 | HE585744 |
| 252 | *T. g. marokkensis* | 86 | M-EH2a | Morocco | Harcha-Oulmés | Graciá et al., 2017 | HE585743 |
| 253 | *T. g. marokkensis* | 87 | M-JK1 | Morocco | south Bab Taza | Graciá et al., 2017 | HE585742 |
| 254 | *T. g. marokkensis* | 87 | 5423 | Morocco | Oulmes | Graciá et al., 2017 | HE585741 |
| 255 | *T. g. marokkensis* | 88 | 5470 | Morocco | Ain Bouali | Graciá et al., 2017 | HE585740 |
| 256 | *T. g. marokkensis* | 89 | 5469 | Morocco | Ain Bouali | Graciá et al., 2017 | HE585739 |
| 257 | *T. g. marokkensis* | 90 | 5445 | Morocco | Ain Bouali | Graciá et al., 2017 | HE585738 |
| 258 | *T. g. nabeulensis* | 91 | 6451 | Libya | unknown exact origin | Graciá et al., 2017 | HE585712 |
| 259 | *T. g. nabeulensis* | 92 | 6460 | Libya | unknown exact origin | Graciá et al., 2017 | HE585711 |
| 260 | *T. g. armenaica* | 9 | DB12518 | Armenia | Nrnadzor | Mashkaryan et al. 2013 | HF954159 |
| 261 | *T. g. armenaica* | 12 | DB12453 | Armenia | Nrnadzor | Mashkaryan et al. 2013 | HF954158 |
| 262 | *T. g. ibera* | 10 | 5614 | Armenia | Nrnadzor | Mashkaryan et al. 2013 | HF954157 |
| 263 | *T. g. armenaica* | 12 | DB12394 | Armenia | Nrnadzor | Mashkaryan et al. 2013 | HF954156 |
| 264 | *T. g. armenaica* | 9 | DB12516 | Armenia | Nrnadzor | Mashkaryan et al. 2013 | HF954155 |
| 265 | *T. g. armenaica* | 9 | DB7811 | Nagorno Karabakh | Hadrut: Aknyakbur | Mashkaryan et al. 2013 | HF954153 |
| 266 | *T. g. armenaica* | 9 | AC211 | Nagorno Karabakh | Kashatagh: Tcobi | Mashkaryan et al. 2013 | HF954145 |
| 267 | *T. g. armenaica* | 9 | DB7821 | Nagorno Karabakh | Kashatagh: Tcobi | Mashkaryan et al. 2013 | HF954144 |
| 268 | *T. g. armenaica* | 12 | Ac209 | Nagorno Karabakh | Kashatagh: Tcobi | Mashkaryan et al. 2013 | HF954143 |
| 269 | *T. g. armenaica* | 12 | Ac208 | Nagorno Karabakh | Kashatagh: Tcobi | Mashkaryan et al. 2013 | HF954142 |
| 270 | *T. g. armenaica* | 93 | AC207 | Nagorno Karabakh | Kashatagh: Tcobi | Mashkaryan et al. 2013 | HF954141 |
| 271 | *T. g. armenaica* | 12 | AC206 | Armenia | Ararat: Urtsadzor | Mashkaryan et al. 2013 | HF954136 |
| 272 | *T. g. armenaica* | 12 | AC205 | Armenia | Ararat: Urtsadzor | Mashkaryan et al. 2013 | HF954135 |
| 273 | *T. g. ibera* | 94 | Ac204 | Armenia | Armavir | Mashkaryan et al. 2013 | HF954126 |
| 274 | *T. g. ibera* | 94 | AC203 | Armenia | Armavir | Mashkaryan et al. 2013 | HF954125 |
| 275 | *T. g. armenaica* | 12 | AC202 | Armenia | Armavir | Mashkaryan et al. 2013 | HF954124 |
| 276 | *T. g. armenaica* | 12 | AC200 | Armenia | Armavir | Mashkaryan et al. 2013 | HF954123 |
| 277 | *T. g. zarudnyi* | 5 | 5275Rab | Iran | Rabor | Javanbakht et al.2017 | KY392866 |
| 278 | *T. g. zarudnyi* | 5 | 5274Rab | Iran | Rabor | Javanbakht et al.2017 | KY392865 |
| 279 | *T. g. zarudnyi* | 3 | 5551Nir | Iran | Nir | Javanbakht et al.2017 | KY392864 |
| 280 | *T. g. zarudnyi* | 3 | 5550Nir | Iran | Nir | Javanbakht et al.2017 | KY392863 |
| 281 | *T. g. zarudnyi* | 3 | 5549Nir | Iran | Nir | Javanbakht et al.2017 | KY392862 |
| 282 | *T. g. zarudnyi* | 5 | 5270Baf | Iran | Baft | Javanbakht et al.2017 | KY392861 |
| 283 | *T. g. zarudnyi* | 3 | 5302Baf | Iran | Baft | Javanbakht et al.2017 | KY392860 |
| 284 | *T. g. zarudnyi* | 5 | 5301Baf | Iran | Baft | Javanbakht et al.2017 | KY392859 |
| 285 | *T. g. buxtoni* | 95 | 5553Na | Iran | Nahoj | Javanbakht et al.2017 | KY392858 |
| 286 | *T. g. buxtoni* | 16 | 5546Ku | Iran | Kuzerash | Javanbakht et al.2017 | KY392857 |
| 287 | *T. g. buxtoni* | 16 | 5544Ku | Iran | Kuzerash | Javanbakht et al.2017 | KY392856 |
| 288 | *T. g. buxtoni* | 96 | 5540Si | Iran | Siyahdare | Javanbakht et al.2017 | KY392855 |
| 289 | *T. g. buxtoni* | 1 | 5315Ni | Iran | Niyaz | Javanbakht et al.2017 | KY392854 |
| 290 | *T. g. buxtoni* | 1 | 5314Ah | Iran | Ahar | Javanbakht et al.2017 | KY392853 |
| 291 | *T. g. buxtoni* | 95 | 5307Is | Iran | Islamabad | Javanbakht et al.2017 | KY392852 |
| 292 | *T. g. buxtoni* | 97 | 5543Su | Iran | Sultanabad | Javanbakht et al.2017 | KY392851 |
| 293 | *T. g. buxtoni* | 98 | 5542Su | Iran | Sultanabad | Javanbakht et al.2017 | KY392850 |
| 294 | *T. g. buxtoni* | 99 | 5541Su | Iran | Sultanabad | Javanbakht et al.2017 | KY392849 |
| 295 | *T. g. buxtoni* | 100 | 5285sh | Iran | Shahrekord | Javanbakht et al.2017 | KY392848 |
| 296 | *T. g. buxtoni* | 101 | 5287sh | Iran | Shahrekord | Javanbakht et al.2017 | KY392847 |
| 297 | *T. g. buxtoni* | 102 | 5280se | Iran | Sepidan | Javanbakht et al.2017 | KY392846 |
| 298 | *T. g. buxtoni* | 1 | 5292na | Iran | Nazarabad | Javanbakht et al.2017 | KY392845 |
| 299 | *T. g. buxtoni* | 1 | 5290na | Iran | Nazarabad | Javanbakht et al.2017 | KY392844 |
| 300 | *T. g. buxtoni* | 103 | 5293na | Iran | Nazarabad | Javanbakht et al.2017 | KY392843 |
| 301 | *T. g. buxtoni* | 95 | 5328Ma | Iran | Mahidasht | Javanbakht et al.2017 | KY392842 |
| 302 | *T. g. buxtoni* | 95 | 5552Ko | Iran | Kordabad | Javanbakht et al.2017 | KY392841 |
| 303 | *T. g. buxtoni* | 101 | 5289kh | Iran | Khomain | Javanbakht et al.2017 | KY392840 |
| 304 | *T. g. buxtoni* | 101 | 5288kh | Iran | Khomain | Javanbakht et al.2017 | KY392839 |
| 305 | *T. g. buxtoni* | 95 | 5298Kal | Iran | Kaleji | Javanbakht et al.2017 | KY392838 |
| 306 | *T. g. buxtoni* | 95 | 5299Kal | Iran | Kaleji | Javanbakht et al.2017 | KY392837 |
| 307 | *T. g. buxtoni* | 104 | 5297Kal | Iran | Kaleji | Javanbakht et al.2017 | KY392836 |
| 308 | *T. g. buxtoni* | 103 | 5282Jol | Iran | Jolfa | Javanbakht et al.2017 | KY392835 |
| 309 | *T. g. buxtoni* | 103 | 5278div | Iran | Divandare | Javanbakht et al.2017 | KY392834 |
| 310 | *T. g. buxtoni* | 103 | 5277div | Iran | Divandare | Javanbakht et al.2017 | KY392833 |
| 311 | *T. g. buxtoni* | 103 | 5276div | Iran | Divandare | Javanbakht et al.2017 | KY392832 |
| 312 | *T. g. buxtoni* | 103 | 5409div | Iran | Divandare | Javanbakht et al.2017 | KY392831 |
| 313 | *T. g. buxtoni* | 95 | 5331Deh | Iran | Dehlili | Javanbakht et al.2017 | KY392830 |
| 314 | *T. g. buxtoni* | 95 | 5332Deh | Iran | Dehlili | Javanbakht et al.2017 | KY392829 |
| 315 | *T. g. buxtoni* | 95 | 5300Baf | Iran | Baft | Javanbakht et al.2017 | KY392828 |
| 316 | *T. g. buxtoni* | 102 | 5326Ars | Iran | Arsanjan | Javanbakht et al.2017 | KY392827 |
| 317 | *T. g. buxtoni* | 95 | 5296ali | Iran | Aliabad | Javanbakht et al.2017 | KY392826 |
| 318 | *T. g. buxtoni* | 95 | 5294ali | Iran | Aliabad | Javanbakht et al.2017 | KY392825 |
| 319 | *T. g. buxtoni* | 103 | 5295ali | Iran | Aliabad | Javanbakht et al.2017 | KY392824 |
| 320 | *T. g. armenaica* | 12 | 5306Agh | Iran | Aghchay | Javanbakht et al.2017 | KY392823 |
| 321 | *T. g. armenaica* | 12 | 5305Agh | Iran | Aghchay | Javanbakht et al.2017 | KY392822 |
| 322 | *T. g. armenaica* | 12 | 5304Agh | Iran | Aghchay | Javanbakht et al.2017 | KY392821 |
| 323 | *T. g. armenaica* | 12 | 5303Jolf | Iran | Jolfa | Javanbakht et al.2017 | KY392820 |
| 324 | *T. g. armenaica* | 12 | 5283Jolf | Iran | Jolfa | Javanbakht et al.2017 | KY392819 |
| 325 | *T. g. terrestris* | 105 | CAS 218245 | Turkey | unknown exact origin | Parham et al. 2006b | DQ080050 |
| 326 | *T. g. nabeulensis* | 106 | MVZ 235707 | Turkey | unknown exact origin | Parham et al. 2006b | DQ080049 |
| 327 | *T. g. nabeulensis* | 107 | TD 5703 | Italy | Sardinia: Mal di Ventre Island | Vamberger et al. 2011 | FR686466 |
| 328 | *T. g. soussensis* | 108 | TD 4437 | Morocco | Souss Valley, Admine Forest | Fritz,U. et al.2009 | FM162043 |
| 329 | *T. g. soussensis* | 109 | TD 4440 | Morocco | Souss Valley, Admine Forest | Fritz,U. et al.2009 | FM162042 |
| 330 | *T. g. soussensis* | 110 | TD 4428 | Morocco | Tafrayate (Tafroute) | Fritz,U. et al.2009 | FM162041 |
| 331 | *T. g. soussensis* | 111 | TD 3826 | Morocco | Aït-Ourir | Fritz,U. et al.2009 | FM162040 |
| 332 | *T. g. soussensis* | 112 | TD 4449 | Morocco | region of Demnate: Iminifri | Fritz,U. et al.2009 | FM162039 |
| 333 | *T. g. soussensis* | 112 | TD 4431 | Morocco | Essaouira | Fritz,U. et al.2009 | FM162038 |
| 334 | *T. g. soussensis* | 112 | TD 3815 | Morocco | foothills of Jbel Amsitene | Fritz,U. et al.2009 | FM162037 |
| 335 | *T. g. soussensis* | 111 | TD 4439 | Morocco | Souss Valley, Admine Forest | Fritz,U. et al.2009 | FM162036 |
| 336 | *T. g. soussensis* | 113 | TD 3818 | Morocco | approx. 25 km N Marrakech | Fritz,U. et al.2009 | FM162035 |
| 337 | *T. g. cyrenaica* | 114 | TD 5046 | Libya | Slonta | Fritz,U. et al.2009 | FM162034 |
| 338 | *T. g. cyrenaica* | 115 | TD 4496 | Libya | Al-Kouf | Fritz,U. et al.2009 | FM162033 |
| 339 | *T. g. marokkensis* | 87 | TD 4183 | Morocco | Quadrass, S Tetouan | Fritz,U. et al.2009 | FM162032 |
| 340 | *T. g. graeca* | 56 | TD 4447 | Morocco | Moulouya River Mouth | Fritz,U. et al.2009 | FM162031 |
| 341 | *T. g. graeca* | 61 | TD 4451 | Morocco | Debdou | Fritz,U. et al.2009 | FM162030 |
| 342 | *T. g. graeca* | 116 | TD 5207 | Spain | Almeria, Centinares | Fritz,U. et al.2009 | FM162029 |
| 343 | *T. g. graeca* | 68 | TD 4101 | Algeria | Khenchela | Fritz,U. et al.2009 | FM162028 |
| 344 | *T. g. graeca* | 56 | D 46394 | Spain | South-eastern Spain | Fritz,U. et al.2009 | FM162027 |
| 345 | *T. g. graeca* | 56 | TD 5187 | Spain | Murcia: Bas Sur | Fritz,U. et al.2009 | FM162026 |
| 346 | *T. g. nabeulensis* | 117 | TD 5004 | Tunisia | Tabarka | Fritz,U. et al.2009 | FM162025 |
| 347 | *T. g. nabeulensis* | 118 | TD 3250 | Algeria | El Kala | Fritz,U. et al.2009 | FM162024 |
| 348 | *T. g. nabeulensis* | 119 | D 44857 | Tunisia | El Kala | Fritz,U. et al.2009 | FM162023 |
| 349 | *T. g. nabeulensis* | 120 | TD 800 | Tunisia | Nabeul | Fritz,U. et al.2009 | FM162022 |
| 350 | *T. g. nabeulensis* | 121 | D 46397 | Tunisia | Confiscated | Fritz,U. et al.2009 | FM162021 |
| 351 | *T. g. nabeulensis* | 106 | D 44865 | Tunisia | unknown exact origin | Fritz,U. et al.2009 | FM162020 |
| 352 | *T. g. terrestris* | 30 | T/HD 22044 | East Turkey | 10 km N Van | Fritz,U. et al.2005 | AJ888354 |
| 353 | *T. g. terrestris* | 30 | T/HD 22061 | East Turkey | Van Gölü | Fritz,U. et al.2005 | AJ888353 |
| 354 | *T. g. anamurensis* | 122 | T/HD 22032 | South Turkey | Anamurium | Fritz,U. et al.2005 | AJ888347 |
| 355 | *T. g. terrestris* | 123 | T/HD 25718 | Jordan | Jarash | Fritz,U. et al.2005 | AJ888346 |
| 356 | *T. g. terrestris* | 124 | T/HD 25717 | Israel | Tiberias | Fritz,U. et al.2005 | AJ888345 |
| 357 | *T. g. terrestris* | 125 | T/HD25318 | Italy | Italy: Sardinia: Sinis Peninsula | Fritz,U. et al.2005 | AJ888343 |
| 358 | *T. g. nabeulensis* | 126 | T/HD 25716 | Israel | Tiberias | Fritz,U. et al.2005 | AJ888344 |
| 359 | *T. g. graeca* | 68 | T/HD 22070 | Libya | unknown exact origin | Fritz,U. et al.2005 | AJ888341 |
| 360 | *T. g. ibera* | 127 | T/HD Tg817 | Greece | Kos Island (Asia Minor) | Fritz,U. et al.2005 | AJ888352 |
| 361 | *T. g. ibera* | 128 | T/HD 25324 | Greece | Kos Island (Asia Minor) | Fritz,U. et al.2005 | AJ888351 |
| 362 | *T. g. ibera* | 10 | T/HD 22057 | Bulgaria | Albena | Fritz,U. et al.2005 | AJ888350 |
| 363 | *T. g. ibera* | 10 | T/HD 22055 | Bulgaria | Albena | Fritz,U. et al.2005 | AJ888349 |
| 364 | *T. g. graeca* | 56 | T/HD 22072 | Mallorca | N Calvia | Fritz,U. et al.2005 | AJ888342 |
| 365 | *T. g. graeca* | 129 | DO_1474 | Spain | Doñana National Park: El Puntal | Graciá et al., 2017 | LT838801 |
| 366 | *T. g. graeca* | 56 | DO_1309 | Spain | Doñana National Park: El Puntal | Graciá et al., 2017 | LT838800 |

**Table S2.** The Mean (± standard deviation) of annual precipitation and temperature seasonality of *T. graeca* from four populations in the contact zone of clades 1 and 2.

| Code | Area | Sample size | Annual precipitation(mm) | Temperature seasonality (SD*100) |
| --- | --- | --- | --- | --- |
| CZ | Central part of Zagros Eco-region | 20 | 192.45±67.32 | 863.55±16.11 |
| SZ | Southern part of Zagros Eco-region | 31 | 250.39±32.70 | 833.82±28.17 |
| P. I-T | Plain areas of Irano-Turanian Eco-region | 16 | 79.65±17.97 | 909.27±14.62 |
| M. I-T | Mountainous areas of Irano-Turanian Eco-region | 15 | 148.41±31.92 | 820.89±31.98 |

**Table S3.** Regression coefficients of morphometric variables for *T. graeca* populations in the contact zone of Clades 1 and 2, using discriminant function. Plastron length (PL), carapace width (CW), maximum carapace width (MCW), carapace height (CH), length of bridge (LB), gular scale length (MGSL), gular scale width (MGSW), humeral scale width (CHSW); maximum pectoral scale width (CPSW), maximum abdominal scale width (CAbSW), maximum femoral scale suture length width (CFSW), maximum anal scale width (CSAW), gular suture length (GSL), Humeral suture length (HSL), pectoral suture length (PSL), abdominal suture length (AbSL), femoral suture length (FSL), anal suture length (ASL), nuchal length (NL), nuchal width (NW), width of first to fifth vertebral scale (VW1-VW5), length of first to fifth vertebral scale (VL1-VL5), dorsal width of supracaudal scale (DSW), ventral width of supracaudal (VSW), supracaudal length (SL), length of first to fourth costal scale (CL1-CL4), and inner height of anterior shell opening parallel to median axis (IHASO). Population codes are given in Table S2.

|  | Populations | | | |
| --- | --- | --- | --- | --- |
| Variable | CZ | SZ | P.I-T | M.I-T |
| constant | -2968.4 | -2836.2 | -2767.4 | -2938 |
| PL | 19.6 | 18.4 | 20.8 | 19.2 |
| CW | -17.2 | -17.2 | -15.6 | -11.5 |
| MCW | 118.8 | 111.8 | 111.5 | 114.2 |
| CH | -55.7 | -59.8 | -56.2 | -61.4 |
| LB | -25.9 | -22.6 | -23.3 | -21.1 |
| MGSL | 164.1 | 163 | 164.7 | 165 |
| MGSW | 39.3 | 37.6 | 31 | 36.9 |
| CHSW | 82.1 | 77 | 73.1 | 79.1 |
| CPSW | -23 | -21.8 | -19.6 | -22.4 |
| CAbSW | -34.9 | -37.6 | -33.5 | -42.8 |
| CFSW | -166.4 | -155.7 | -158 | -159.1 |
| CSAW | 119.5 | 117.8 | 116.2 | 121.4 |
| GSL | -85.2 | -85.6 | -84.2 | -87.6 |
| HSL | 14.9 | 15.1 | 17.4 | 12.9 |
| PSL | 120.6 | 120.6 | 119.3 | 126.4 |
| AbSL | 90.8 | 90.9 | 91.3 | 89 |
| FSL | 46 | 53.7 | 40.7 | 47.5 |
| ASL | 25.3 | 28.6 | 21.2 | 21.4 |
| NL | 51.9 | 53.5 | 60.4 | 46.3 |
| NW | 50.3 | 52.9 | 48.4 | 53.6 |
| VW1 | 66.1 | 65.4 | 65.9 | 68.3 |
| VW2 | -93.6 | -89.8 | -90.8 | -83.7 |
| VW3 | 80.9 | 83.9 | 85.4 | 87.6 |
| VW4 | 41.4 | 36.1 | 34.4 | 30.6 |
| VW5 | 97.7 | 100.4 | 99.7 | 112.2 |
| VL1 | 9.5 | 4.9 | -0.7 | 2.4 |
| VL2 | -48.7 | -43.9 | -42.6 | -38.6 |
| VL3 | 15 | 15 | 15 | 16.7 |
| VL4 | 4 | 1.9 | 1.2 | 4.3 |
| VL5 | -31.9 | -35.6 | -32.8 | -43.6 |
| DSW | 22.1 | 21.9 | 18.3 | 21.7 |
| VSW | -15.9 | -18.3 | -16.3 | -18.9 |
| SL | 64 | 66.5 | 66.5 | 69 |
| CL1 | 110.7 | 113.5 | 110.1 | 118.2 |
| CL2 | 197.4 | 186 | 185.5 | 186.2 |
| CL3 | 119 | 121.3 | 109.1 | 116.3 |
| CL4 | 12.9 | 16.3 | 7.5 | 10.2 |
| IHASO | 85.9 | 86.5 | 85.4 | 87.6 |
